# HERC2 inactivation abrogates nucleolar localization of RecQ helicases BLM and WRN

**DOI:** 10.1038/s41598-020-79715-y

**Published:** 2021-01-11

**Authors:** Mingzhang Zhu, Wenwen Wu, Yukiko Togashi, Weixin Liang, Yasuo Miyoshi, Tomohiko Ohta

**Affiliations:** 1 Department of Translational Oncology, St. Marianna University Graduate School of Medicine, 2-16-1, Sugao, Miyamae-ku, Kawasaki, 216-8511 Japan; 2 Department of General Surgery, The People’s Hospital of Gaoming District of Foshan City, Foshan, 528500 Guangdong China; 3 Division of Breast and Endocrine Surgery, Department of Surgery, Hyogo College of Medicine, Hyogo, 663-8501 Japan

**Keywords:** Cancer genomics, Cancer therapy, Nuclear organization, Organelles, DNA damage and repair, DNA replication, Ribozymes

## Abstract

The nucleolus is a nuclear structure composed of ribosomal DNA (rDNA), and functions as a site for rRNA synthesis and processing. The rDNA is guanine-rich and prone to form G-quadruplex (G4), a secondary structure of DNA. We have recently found that HERC2, an HECT ubiquitin ligase, promotes BLM and WRN RecQ DNA helicases to resolve the G4 structure. Here, we report the role of HERC2 in the regulation of nucleolar localization of the helicases. Furthermore, HERC2 inactivation enhances the effects of CX-5461, an inhibitor of RNA polymerase I (Pol I)-mediated transcription of rRNA with an intrinsic G4-stabilizing activity. HERC2 depletion or homozygous deletion of the C-terminal HECT domain of HERC2 prevented the nucleolar localization of BLM and WRN, and inhibited relocalization of BLM to replication stress-induced nuclear RPA foci. HERC2 colocalized with fibrillarin and Pol I subunit RPA194, both of which are required for rRNA transcription. The HERC2 dysfunction enhanced the suppression of pre-rRNA transcription by CX-5461. These results suggest the effect of HERC2 status on the functions of BLM and WRN on rRNA transcription in the nucleolus. Since HERC2 is downregulated in numerous cancers, this effect may be clinically relevant considering the beneficial effects of CX-5461 in cancer treatments.

## Introduction

The nucleolus is a non-membranous nuclear organelle, which is involved in ribosome biogenesis and is composed of ribosomal DNA (rDNA). It serves as a site for rRNA synthesis and processing for ribosome assembly^1,2^. In addition, the nucleolus is critical in the cellular response to DNA damage, and incorporates substantial range of DNA damage repair proteins, including RecQ DNA helicases BLM and WRN^2–5^. While these proteins reside in the nucleolus as cellular stock for the damage response, they also plays critical roles in the rRNA synthesis^2–5^.

Guanine-rich DNA regions, such as rDNA, telomeres, and the promoter sequences, are prone to form G-quadruplex (G4), a secondary structure of DNA^6–9^. G4s are stacked structures built with DNA motifs containing four stretches of three or more consecutive guanines via Hoogsteen base pairing stabilized by a monovalent cation^10,11^. Although G4s play essential physiological roles, they may interrupt the process of replication or transcription if left unresolved. To overcome these obstacles, cells harbor G4 unwinding helicases, such as PIF1, DNA2, FANCJ, DDX11, RTEL1, RHAU/DHX36, BLM and WRN.

Each helicase plays an essential role in the resolution of G4 secondary structures. BLM, WRN and RHAU are involved in unwinding of noncanonical DNA structures in a 3′ to 5′ direction^12^, whereas PIF1, DNA2, FANCJ, DDX11 and RTEL1 are 5′ to 3′ helicases^13^. In addition to G4s, BLM resolves a variety of DNA substrates including 3′-tailed duplexes, bubble structures, forked duplexes, DNA displacement loops, and double Holliday junctions^12,14^, to prevent inappropriate recombination and to resolve stalled replication forks^15–17^. Similar to BLM, WRN plays essential roles in DNA repair and replication by disrupting G4s, bubble structures, and double Holliday junctions at perturbed replication forks or DNA double-strand breaks^12,17–19^. In addition to the helicase activity, WRN possesses an exonuclease activity^20^. Mutations in BLM and WRN result in multiple deleterious effects associated with cancer, called chromosome instability syndromes, such as Bloom and Werner syndromes, respectively^18,21^ .

BLM and WRN, as well as their yeast orthologue Sgs1, are localized in the nucleolus and carry out essential and fundamental functions in the nucleolus^22–26^. BLM interacts with RNA polymerase I (Pol I), and is involved in pre-rRNA transcription owing to its helicase activity^24^. BLM-deficient cells show reduced pre-rRNA transcription accompanied by a decrease in mature 18S and 28S rRNA. Additionally, WRN interacts with Pol I and depletion of WRN results in decreased levels of rRNA transcription and ribosomal subunits^25,26^.

Although the roles of BLM and WRN in the nucleolus and their re-localization in response to DNA damage have been intensively studied, the regulatory mechanisms underlying their localization remain poorly understood. In one of our recent studies, BLM and WRN were identified as interactors of HERC2, a large HECT-type ubiquitin E3 ligase implicated in DNA replication and damage response, via mass-spectrometric analyses of the HERC2 complex^27^. We have demonstrated that HERC2 epistatically regulates BLM and WRN to resolve the G4 structure by promoting the assembly of replication protein A (RPA) on helicase complexes of BLM and WRN^27,28^. Inactivation of HERC2 resulted in G4 accumulation and lead cells to hypersensitive to G4 stabilizers such as pyridostatin and telomestatin^27,29^. In this study, we aimed to investigate the role of HERC2 in the regulatory mechanisms underlying nucleolar localization of BLM and WRN.

## Results

### HERC2 is required for nucleolar localization of BLM and WRN

**Figure 1:**
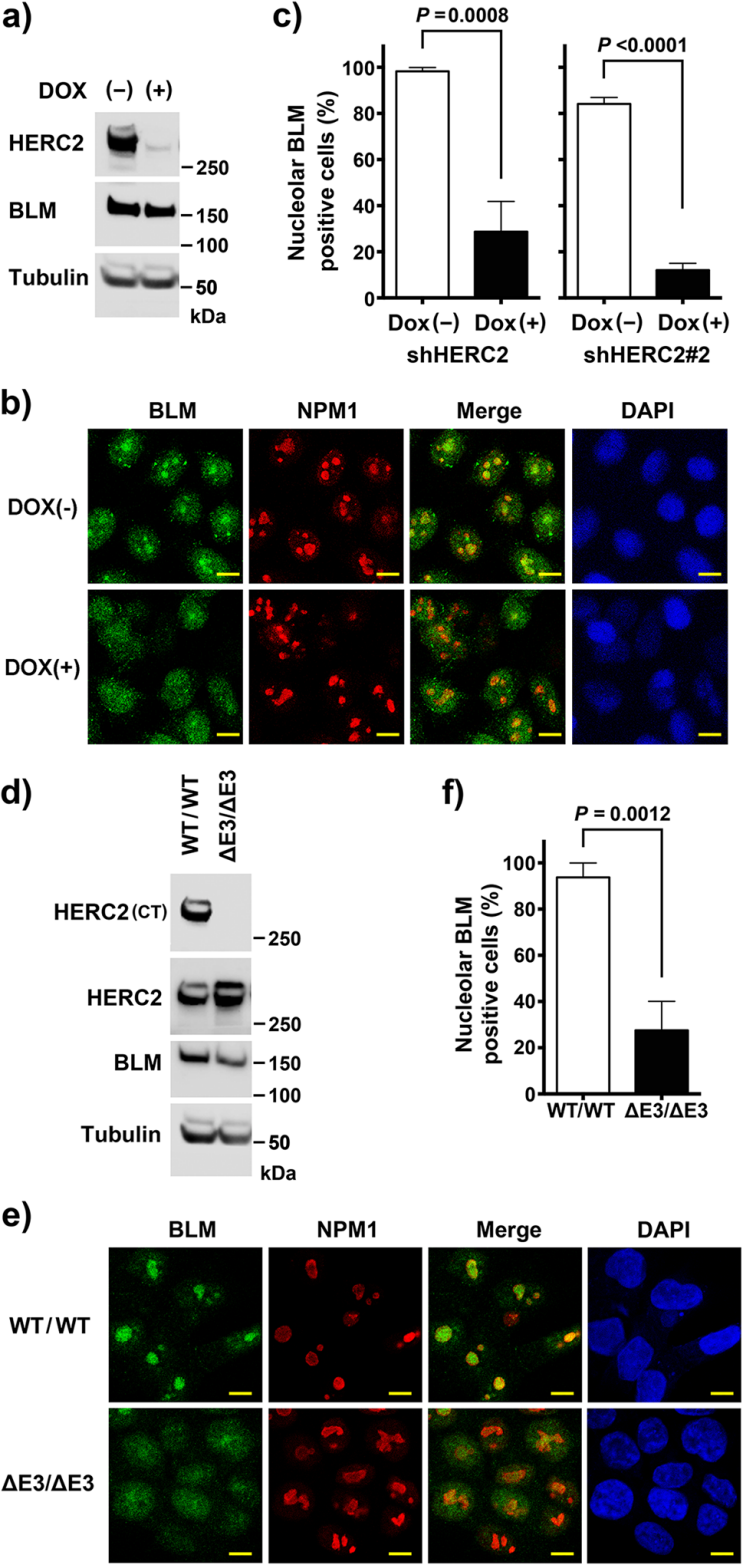
Nucleolar localization of BLM requires HERC2 and its E3 domain. **(a,b)** HeLa-shHERC2 cells, with or without Dox-mediated induction, were subjected to immunoblotting **(a)** or immunostain **(b)** with the indicated antibodies. The nuclei were counter stained with DAPI. Scale bar, 10 μm. **(c)** Quantifications of the nucleolar BLM positive cell from **(b)** (left panel), and those from HeLa cells with a different shRNA (shHERC2#2, right panel, see also Supplementary Figure S1) are shown. Error bars, S.D. of three independent experiments, each based on more than 100 cells. Statistical significances was calculated using the Student’s *t-*test. **(d,e)** Wild-type or HERC2^ΔE3/ΔE3^ HCT116 cells were subjected to immunoblotting **(d)** or immunostaining **(e)** with the indicated antibodies. Antibodies HERC2 (CT) and HERC2 recognize residues 4389–4834 and 1781–1974 of HERC2, respectively. Scale bar, 10 μm. **(f)** Quantification of the nucleolar BLM positive cell. Error bars, S.D. of three independent experiments, each based on more than 100 cells. Statistical significances was calculated using the Student’s *t-*test. Full-length blots/gels are presented in Supplementary Figure S12.

In our previous study, we have demonstrated that HERC2 interacts with BLM and regulates its functions^27,28^. To investigate the regulatory role of HERC2 on the cellular localization of BLM, we analyzed the effect of HERC2 depletion on BLM localization. HeLa cells stably expressing doxycycline (Dox)-inducible shRNA against HERC2 (HeLa-shHERC2) were subjected to Dox-mediated induction or were not induced with Dox, and an effective depletion was confirmed via immunoblotting (Fig. 1A). Using the cells, we co-immunostained BLM with nucleophosmin (NPM1, also known as B23), a marker for the nucleolus, as BLM has been reported to localize in the nucleolus of unstressed cells especially with high incidence in the S phase^23^, the period when the HERC2-BLM interaction was observed^27^. Strong BLM signal was observed in the nucleolus in almost all cells without Dox induction, with a higher population of cells expressing the BLM signal than that previously reported^23^. This could be attributed to the criteria of the positivity considered, cell line used, and/or sensitivity of the antibody used. Interestingly, we observed dramatic reduction in the nucleolar localization of BLM in HERC2-depleted cells, compared to that in control cells (28.7% ± 13.1% vs 98.2% ± 1.6%) (Fig. 1B,C). Similar results were also observed in cells with a different shRNA targeting an independent sequence in HERC2, arguing against off-target effects (12.1% ± 3.0% vs 84.1% ± 2.8%) (Fig. 1C and Supplementary Figure S1).

Since the inhibition of HERC2 E3 activity was also found to affect the function of BLM, we examined its effect on the BLM nucleolar localization. We used HCT116 cells lacking the C-terminal HERC2 catalytic ubiquitin-binding site as a result of CRISPR/Cas9-mediated insertion of the stop codon at E4758 (HCT116-HERC2^ΔE3/ΔE3^). HERC2 ubiquitinates RPA2 in proliferating cells in the absence of stress^27,28^. However, these effects have not been detected for BLM and WRN (Supplementary Figure S2). HERC2^ΔE3/ΔE3^ can bind to BLM, WRN, and RPA, similar to that observed with the wild-type HERC2; however, it fails to ubiquitinate RPA2^27,28^. The steady-state level observed with HCT116-HERC2^ΔE3/ΔE3^ was approximately the same as that observed with the wild-type, which was indicated by using an antibody against the central epitope of HERC2 (Fig. 1D). HCT116-HERC2^ΔE3/ΔE3^ and wild-type cells were further immunostained with BLM and NPM1. Similar to HERC2-depleted HeLa cells, nucleolar BLM signal was remarkably reduced in HERC2^ΔE3/ΔE3^ cells, compared to that in wild-type cells (27.6% ± 12.5% vs 93.8% ± 6.2%)(Fig. 1E,F).

**Figure 2:**
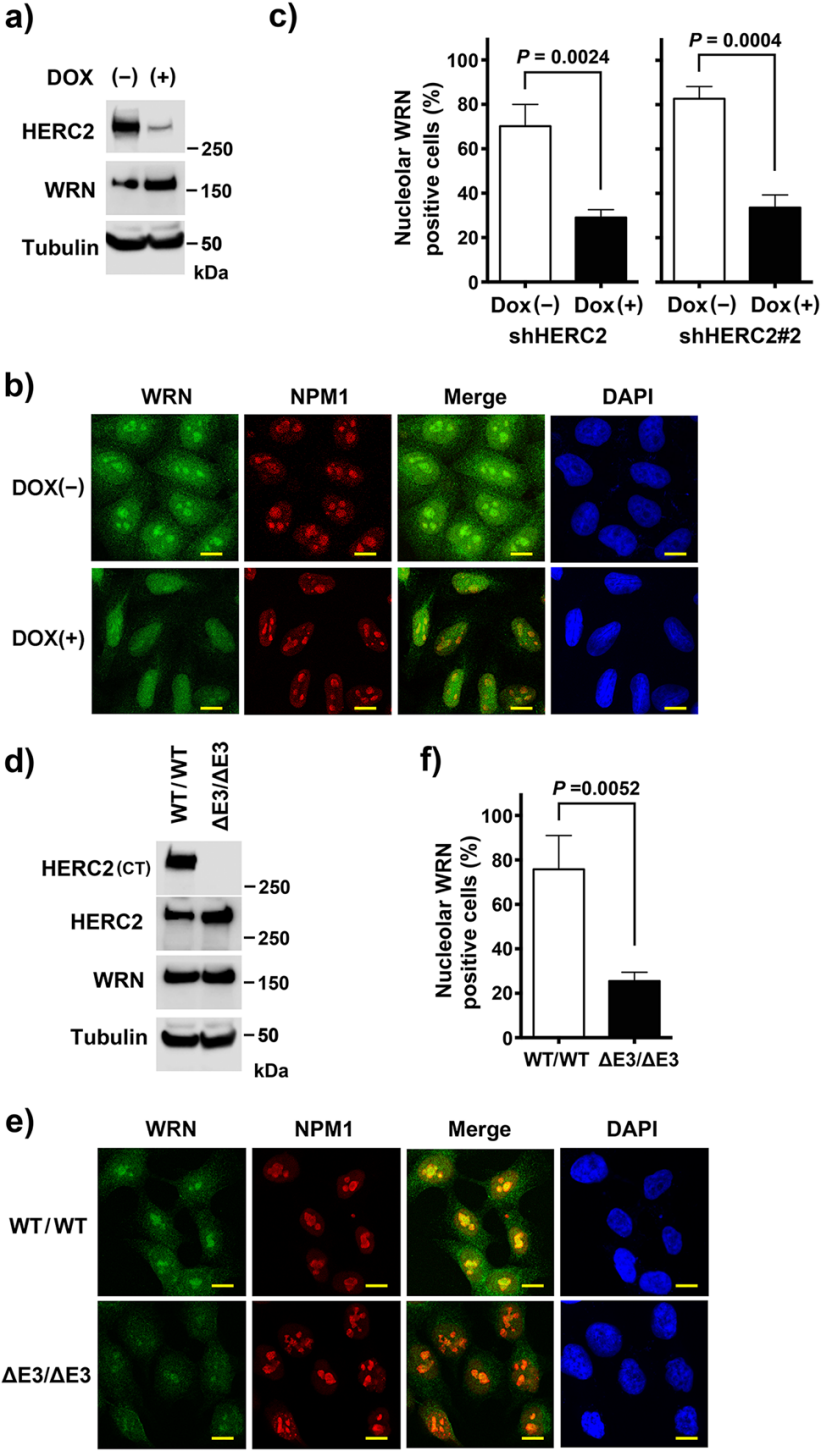
Nucleolar localization of WRN requires HERC2 and its E3 domain. **(a,b)** HeLa-shHERC2 cells, with or without Dox-mediated induction, were subjected to immunoblotting **(a)** or immunostain **(b)** with the indicated antibodies. The nuclei were counter stained with DAPI. Scale bar, 10 μm. **(c)** Quantifications of the nucleolar WRN positive cell from **(b)** (left panel), and those from HeLa cells with a different shRNA (shHERC2#2, right panel, see also Supplementary Figure S1) are shown. Error bars, S.D. of three independent experiments, each based on more than 100 cells. Statistical significances was calculated using the Student’s *t-*test. **(d,e)** Wild-type or HERC2^ΔE3/ΔE3^ HCT116 cells were subjected to immunoblotting **(d)** or immunostain **(e)** with the indicated antibodies. Scale bar, 10 μm. **(f)** Quantification of the nucleolar WRN positive cell. Error bars, S.D. of three independent experiments, each based on more than 100 cells. Statistical significances was calculated using the Student’s *t-*test. Full-length blots/gels are presented in Supplementary Figure S12.

The regulation of BLM nucleolar localization by HERC2 prompted us to investigate its regulatory role in the nucleolar localization of another RecQ helicase called WRN. HeLa-shHERC2 cells were subjected to Dox-mediated induction or were not induced with Dox, and the depletion of HERC2 was confirmed via immunoblotting (Fig. 2A). Nucleolar localization of WRN was analyzed by co-immunostaining with NPM1. WRN signal in the nucleolus was detected in approximately two thirds of cells without Dox induction, and was found to be significantly reduced by HERC2 depletion (70.2% ± 9.8% vs 29.0% ± 3.6%)(Fig. 2B,C). The same effect was observed in HeLa cells with a different shRNA (82.7% ± 5.5% vs 33.6 ± 5.7%) (Fig. 2C and Supplementary Figure S1), and HCT116-HERC2^ΔE3/ΔE3^ cells, compared to that in wild-type cells (75.8% ± 15.2% vs 25.5 ± 4.0%; Fig. 2D–F). It has been reported that HERC2 is an assembly factor for ubiquitin E3 ligase, RNF8, and E2-conjugating enzyme, Ubc13, in response to DNA damage^30^. Furthermore, RNF8 interacts with another DNA damage-responsive E3 ligase, RNF168, to promote the recruitment of BLM to sites of replication fork stalling^31^. Thus, we examined whether the status of RNF8 and RNF168 affected the nucleolar localization of BLM and WRN in normal proliferating cells in the absence of stress. However, we observed that depletion of RNF8 or RNF168 did not affect the nucleolar localization of BLM and WRN (Supplementary Figure S3), suggesting that the observed effect of HERC2 dysfunction was not associated with RNF8 and RNF168.

Together, these results indicate that HERC2 and its E3 ligase activity maintain the nucleolar localization of RecQ helicases BLM and WRN in unstressed cells.

### HERC2 is prerequisite for relocalization of BLM to RPA foci in response to replication stress

**Figure 3:**
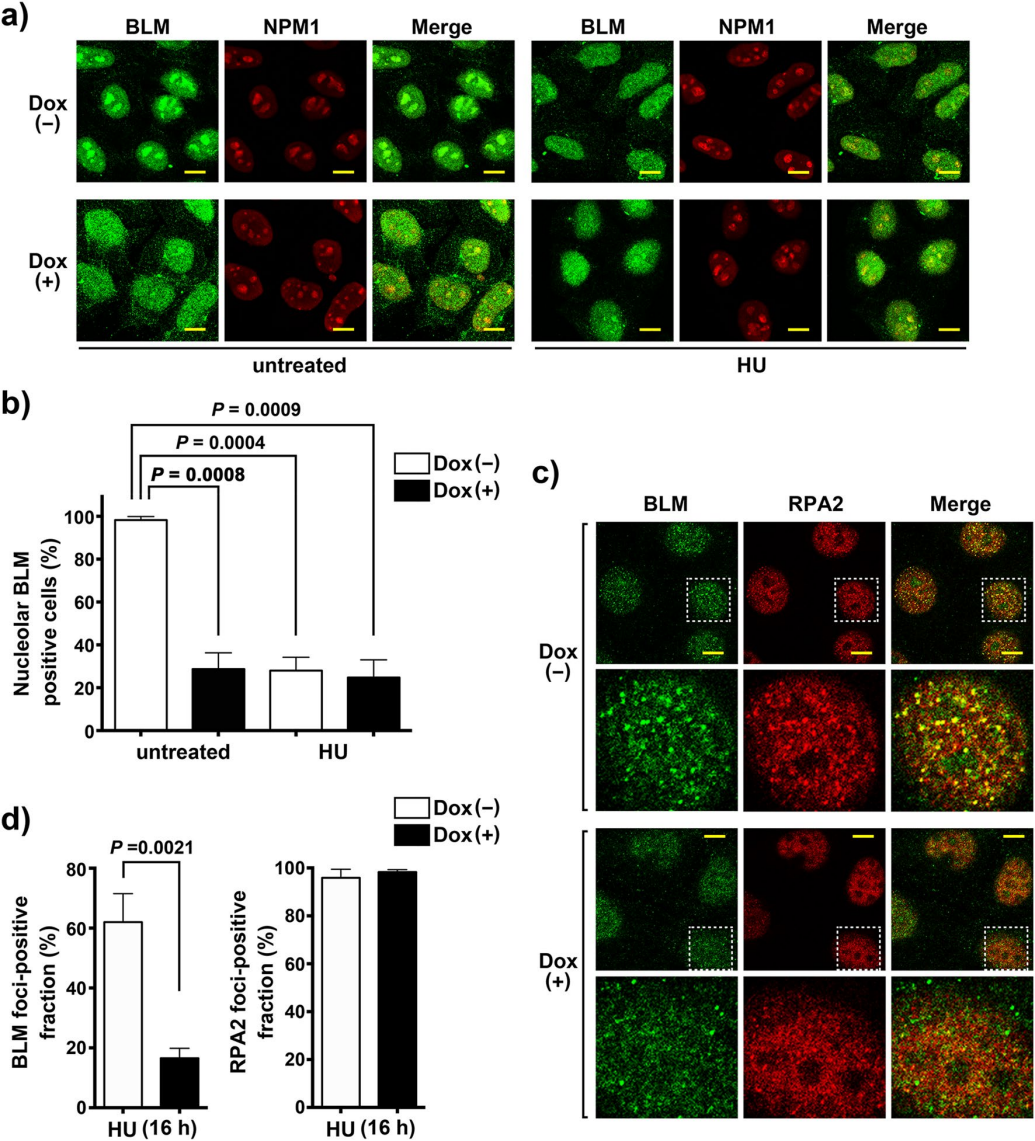
BLM translocation in response to replication stress is dismissed by HERC2 depletion. **(a)** BLM disappears from the nucleolus in response to replication stress. HeLa-shHERC2 cells with or without Dox-mediated induction, untreated or treated with 1 mM HU for 4 h, were immunostained with antibodies against BLM and NPM1. Scale bar, 10 μm. **(b)** Quantification of the nucleolar BLM positive cell with or without HU treatment. Error bars, S.D. of three independent experiments, each based on more than 100 cells. Statistical significances was calculated using the Student’s *t-*test. **(c)** HERC2 is required for relocalization of BLM to RPA2 nuclear foci in response to replication stress. HeLa-shHERC2 cells with or without Dox-mediated induction, treated with 1 mM HU for 16 h, were immunostained with the indicated antibodies. One representative nucleus (dashed line) in the upper panels has been magnified and shown in the lower panels. Colocalization of BLM and RPA2 is shown in the merged display as yellow foci. Scale bar, 10 μm. **(d)** Quantification of the cells displaying more than ten BLM foci co-localizing with RPA2 (left panel) or discrete RPA2 foci (right panel). Error bars, S.D. of three independent experiments, each based on more than 50 cells. Statistical significances were calculated using Student’s *t-*test.

The role of nucleolus in stress response has been well established. It sequestrates a number of proteins that are released in response to various stresses, including DNA damage or replication stress^1,2,32^. In response to the replication stress, BLM is recruited to the stalled replication fork where RPA, a protein complex that binds to single-strand DNA (ssDNA) to prevent the formation of secondary structures, can be detected as discrete nuclear foci^33,34^. Since the nucleolus lacks BLM in HERC2-depleted cells, we examined whether it affected the relocalization of BLM to the stalled replication fork. We first analyzed the nucleolar localization of BLM following treatment with hydroxyurea (HU). HeLa-shHERC2 cells were subjected to Dox-mediated induction or were not induced with Dox, untreated or treated with HU, and the nucleolar localization of BLM was analyzed by co-immunostaining with NPM1. Consistent with previous results^35^, BLM signal in the nucleolus was significantly reduced in cells without Dox after treatment with HU for 4 h (98.2% ± 1.6% vs 28.0 ± 10.8%) (Fig. 3A,B). In contrast, the decreased level of BLM in HERC2-depleted cells was not further reduced by the HU treatment (28.7% ± 13.1% vs 24.8% ± 14.2%). Similar effects of HU treatment in combination with HERC2 depletion were observed on WRN nucleolar localization (Supplementary Figure S4). We then examined the effect of HERC-depletion on the recruitment of BLM to HU-induced stalled replication forks. Time-course analysis showed that co-localization of nuclear BLM foci with RPA2 appeared 10 h after HU addition and was found to be abundant at 16 h (Supplementary Figure S5). Importantly, the co-localization of BLM foci with RPA2 were significantly reduced in Dox-induced HERC2-depleted cells (62.0% ± 5.5% vs 16.6% ± 3.3%, *P* = 0.0021) whereas RPA2 foci were unaffected (Fig. 3C,D, and Supplementary Figure S5). The same effect was observed in HCT116-HERC2^ΔE3/ΔE3^ cells (Supplementary Figure S6). In the case of HCT116-HERC2^ΔE3/ΔE3^ cells, the intensity of RPA2 in nucleoplasm was seemingly higher than that in wild-type cells, possibly due to the lack of HERC2-mediated RPA2 ubiquitination and degradation as previously reported^28^. This resulted in a merged stain of BLM and RPA2 in the nucleoplasm. However, discrete nuclear foci of BLM co-localizing with RPA observed in the WT were found to be reduced in HERC2^ΔE3/ΔE3^ cells.

Altogether, these results indicate that HERC2 plays a crucial role in the relocalization of BLM from the nucleoplasm to the stalled replication fork.

### HERC2, BLM and WRN localizes at the fibrillar center of the nucleolus

**Figure 4:**
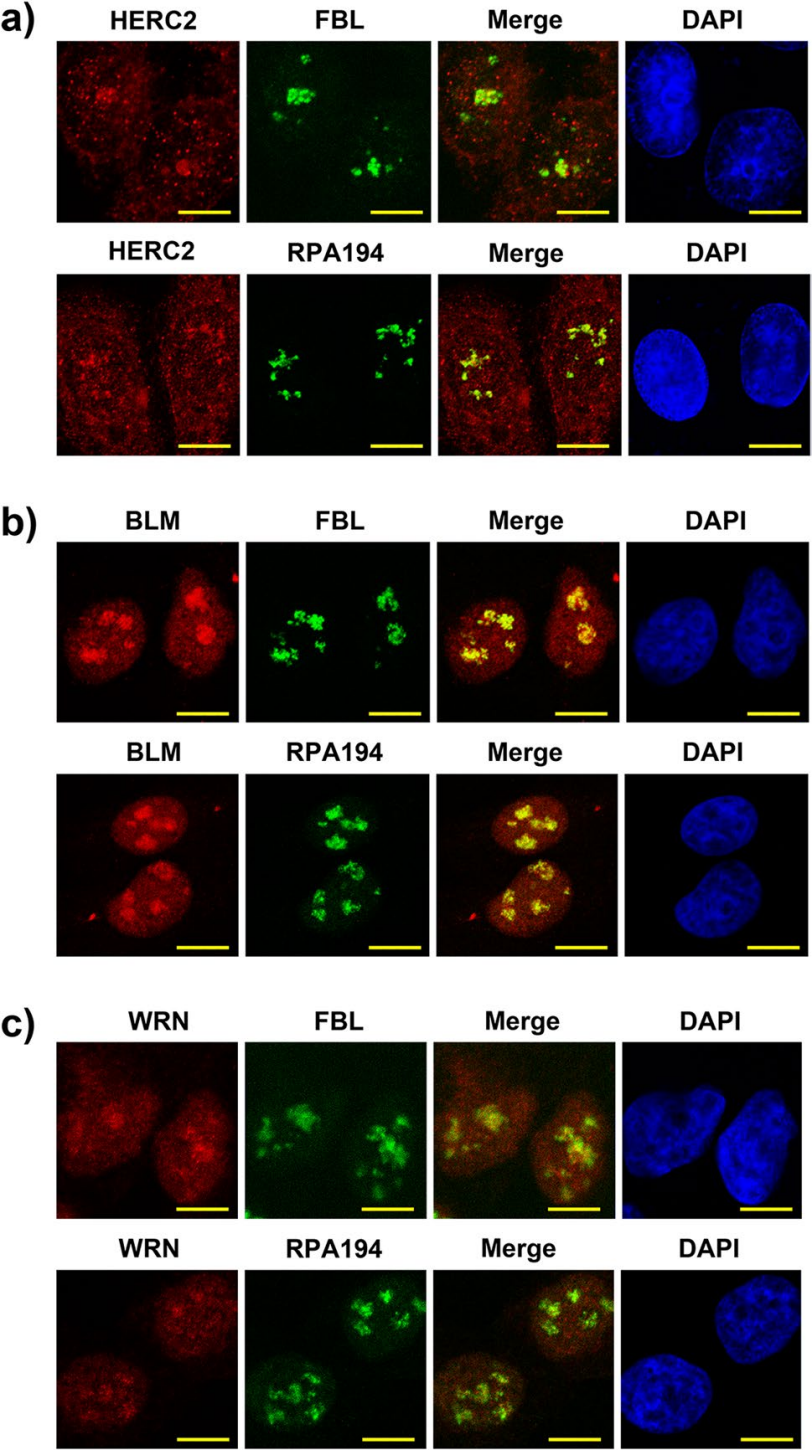
HERC2, BLM and WRN co-localize with FBL and RPA194. Spontaneously growing HeLa cells are subjected to immunostaining with HERC2 **(a)**, BLM **(b)** or WRN **(c)** coimmunostained with FBL (upper panels) or RPA194 (lower panels) as indicated. The nuclei were counter stained with DAPI. Scale bar, 10 μm.

HERC2 has been previously reported to co-localize at the replication fork with RPA and PCNA in unstressed cells^27,36^. In addition to the nuclear foci, we observed that HERC2 also resided in the nucleolus throughout the cell cycle. Upon precise observation of HERC2 stain merged with DAPI, we found that HERC2 localized at the center of the nucleolus where DAPI concentration was low due to increased transcription activity of ribosomal genes (Supplementary Fig. S7). The nucleolus is constituted by three major components, namely the fibrillar center (FC), the dense fibrillar component (DFC), and the granular component (GC), associated with pre-rRNA transcription, pre-rRNA processing, and ribosome assembly, respectively^1,2^. The GC comprises the outer layer of the organelle, whereas FC and DFC constitute the inner regions where Pol I and pre-rRNA processing factors such as fibrillarin (FBL) are present^1,2^. The localization of HERC2 at the center of the nucleolus prompted us to examine whether HERC2 colocalized with the markers of FC and DFC. As shown in Fig. 4A, co-immunostaining of HERC2 with FBL and RPA194, a subunit of Pol I, revealed that these proteins colocalized in the inner region of the nucleolus, suggesting a possible role of HERC2 in rRNA transcription and processing. BLM has been previously reported to be involved in pre-rRNA transcription^24^ and WRN has also been found to interact with Pol I, thereby regulating rRNA transcription^26^. Therefore, we determined their localization in the nucleolus. Immunofluorescence analyses demonstrated that BLM and WRN colocalized with FBL and RPA194, similar to that observed with HERC2 (Fig. 4B,C).

### HERC2 dysfunction enhances the suppressive effect of CX-5461 on pre-rRNA transcription

**Figure 5:**
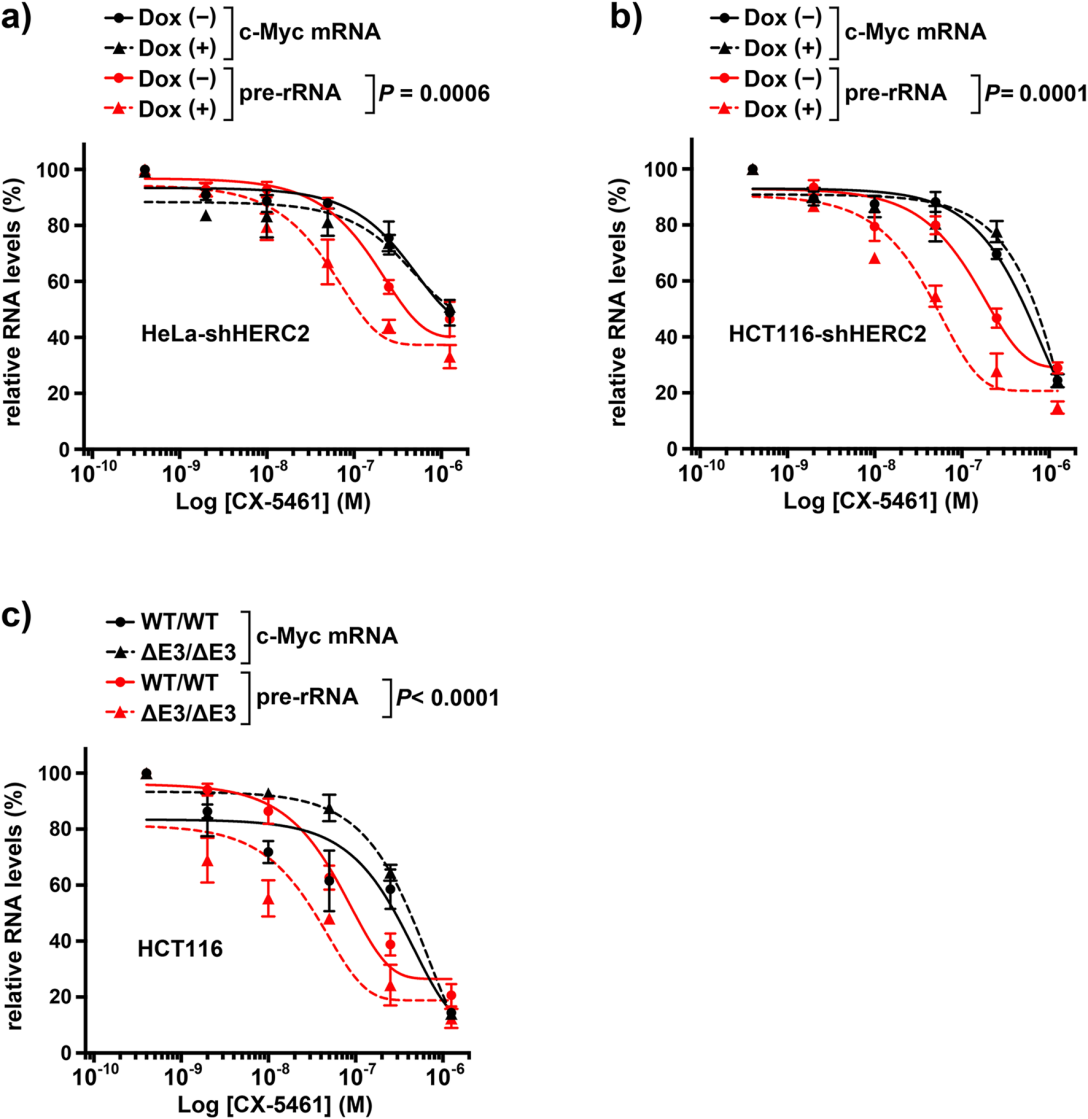
HERC2 dysfunction enhances the inhibitory effect of CX-5461 on pre-rRNA transcription. HeLa-shHERC2 **(a)** or HCT116-shHERC2 **(b)** cells untreated (solid line) or treated with Dox (dashed line), and wild-type (solid line) or HERC2^ΔE3/ΔE3^ (dashed line) HCT116 cells **(c)** were incubated with the indicated dose of CX5461. Inhibition of pre-rRNA (red) and c-Myc mRNA (black) transcription was analyzed by qRT-PCR, and was normalized against β-actin mRNA expression levels (see Supplementary Figure S8 for the expression level of β-actin mRNA). Data are shown as the means ± S.D of three independent experiments. *P*-values of interactions were calculated using two-way ANOVA. The concentration that inhibited 50% of the RNA products (IC50 value) was as follows: **(a)** pre-rRNA Dox (−): 0.77 μmol/l, Dox ( +): 0.21 μmol/l, others: n.a., **(b)** c-Myc-mRNA Dox (−): 0.47 μmol/l, Dox ( +): 0.52 μmol/l, pre-rRNA Dox (−): 0.26 μmol/l, Dox ( +) 0.06 μmol/l, **(c)** c-Myc-mRNA WT/WT: 0.15 μmol/l, ΔE3/ΔE3: 0.35 μmol/l, pre-rRNA WT/WT: 0.13 μmol/l, ΔE3/ΔE3 0.03 μmol/l.

The co-localization of HERC2 with factors for rRNA synthesis and the observed effect of HERC2 on the nucleolar localization of BLM and WRN, helicases critical for rRNA transcription, motivated us to investigate whether HERC2 altered the effect of CX-5461, an inhibitor of Pol I-mediated rRNA synthesis^37^. Therefore, we analyzed the effect of HERC2 dysfunction in CX-5461-induced suppression of pre-rRNA transcription. HeLa-shHERC2 cells were subjected to Dox-mediated induction or were not induced with Dox, and then incubated with different doses of CX-5461. Expression levels of pre-rRNA and *c-Myc* mRNA were determined via qRT-PCR. The expression of pre-rRNA was more significantly suppressed than that of *c-Myc* mRNA (Fig. 5A) in concordance with the findings of a previous report^37^, which suggests relatively specific effect of this agent on the transcription by Pol I compared to that by Pol II. Importantly, depletion of HERC2 significantly reduced the expression of pre-rRNA, whereas it did not affect the expression of *c-Myc* mRNA (Fig. 5A). The same results were recapitulated in HCT116-shHERC2 cells (Fig. 5B) and HCT116-HERC2^ΔE3/ΔE3^ cells (Fig. 5C). These results indicate that HERC2 dysfunction sensitizes cells to CX-5461-mediated suppression of rRNA synthesis. Interestingly, we observed that CX-5461 reduced the nucleolar localization of BLM and WRN (Supplementary Figure S9). This suggests that reduced rDNA transcription by HERC2 inactivation may be responsible for the observed alteration in BLM and WRN localization (Figs. 1 and 2). However, depletion of HERC2 or HERC2^ΔE3/ΔE3^ on its own did not suppress the pre-rRNA expression in the absence of CX-5461 (Supplementary Figure S10). This indicates that HERC2 regulates localization of BLM and WRN independent of the suppression of rDNA transcription.

### HERC2 dysfunction alters the sensitivity of cells to CX-5461

**Figure 6:**
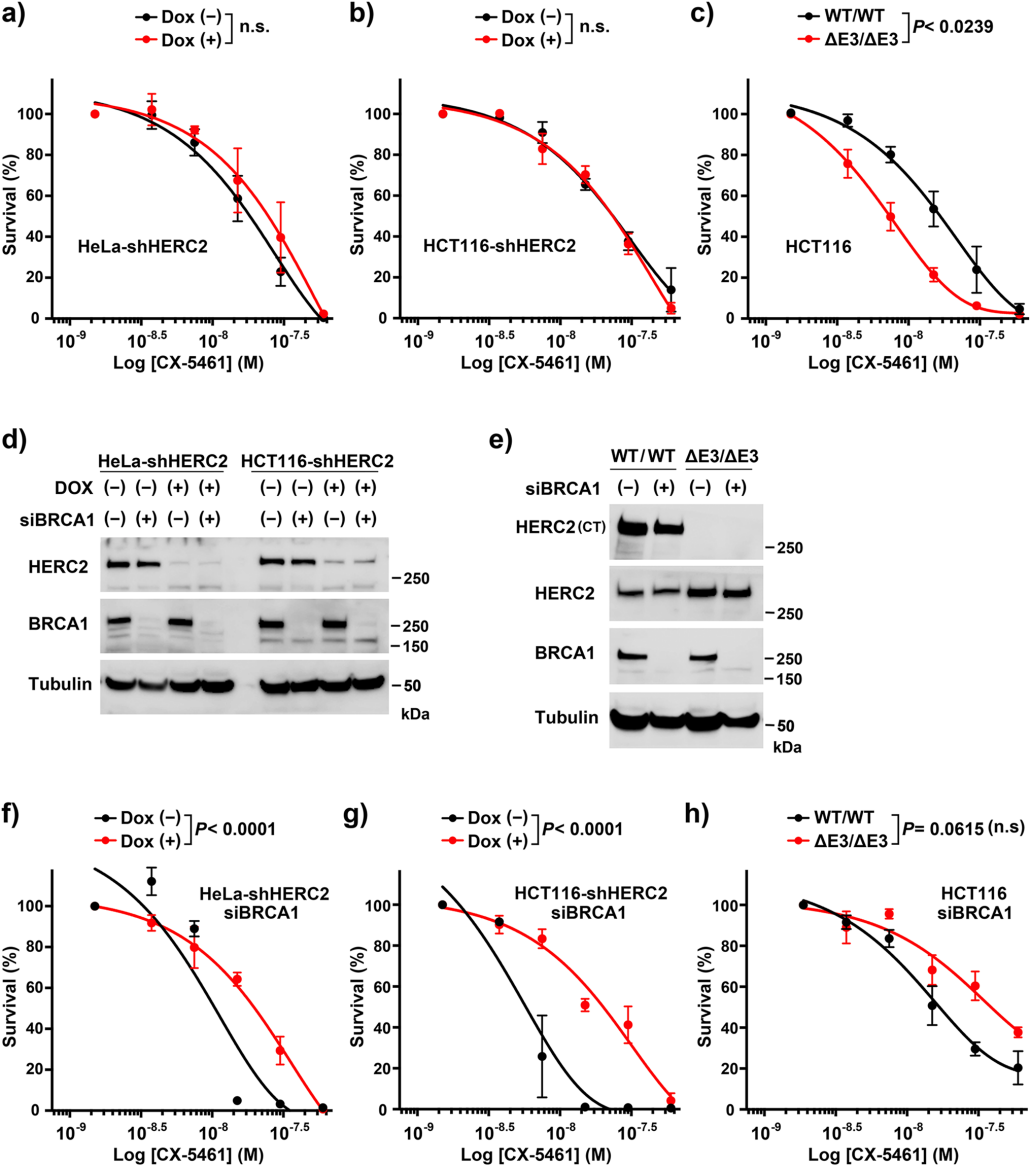
HERC2 dysfunction alters the sensitivity of cells to CX-5461. **(a–c)** HeLa-shHERC2 **(a)** and HCT116-shHERC2 **(b)** cells, with or without Dox-mediated induction, or WT or HERC2^ΔE3/ΔE3^ HCT116 cells **(c)** were exposed to indicated doses of the CX5461 for 24 h and analyzed for clonogenic survival after two weeks. The data are shown with the nonlinear regression fit curves of one phase decay (GraphPad Prism). **(d–h)** Cells as in **(a–c)** were transfected with control (–) or BRCA1-specific siRNA, with or without Dox-mediated induction, and either subjected to immunoblotting with the indicated antibodies **(d,e)** or clonogenic survival assays (**f–h**, and supplementary Figure S11) as in **(a–c)**. Average ± SD values, normalized to cells without agents were derived from three independent experiments. *P*-values of interactions were calculated using two-way ANOVA. The concentration that inhibited 50% of the colonies (IC50 value) was as follows: **(a)** Dox (−): 17.3 nmol/l, Dox ( +): 22.3 nmol/l, **(b)** Dox (−): 22.4 nmol/l, Dox ( +): 21.6 nmol/l, **(c)** WT/WT: 16.1 nmol/l, ΔE3/ΔE3: 7.3 nmol/l, **(f)** Dox (−): 10.0 nmol/l, Dox ( +): 18.3 nmol/l, **(g)** Dox (−): 6.1 nmol/l, Dox ( +): 18.1 nmol/l, **(h)** WT/WT: 17.8 nmol/l, ΔE3/ΔE3: 39.3 nmol/l. Full-length blots/gels are presented in Supplementary Figure S12.

In addition to inhibition of Pol I activity, CX-5461 possesses an intrinsic activity as a G4 stabilizer^38^. CX-5461 also promotes topoisomerase II trapping possibly following G4 stabilization^39,40^. We have previously reported that HERC2 suppresses G4 formation of DNA in a manner which is epistatic to BLM and WRN. Furthermore, HERC2 dysfunction leads to G4 accumulation and sensitizes cells to G4 stabilizers pyridostatin and telomestatin^27,29^. Therefore, we hypothesized that HERC2 dysfunction could also sensitize cells to CX-5461. HeLa-shHERC2 and HCT116-shHERC2 cells, subjected to Dox-mediated induction or without induction, or exponentially growing wild-type and HCT116-HERC2^ΔE3/ΔE3^ cells were exposed to different doses of CX5461, and allowed to form colonies. However, in contrast to our expectations, Dox-induced depletion of HERC2 did not sensitize the cells to CX5461 (Fig. 6A,B), whereas HCT116 HERC2^ΔE3/ΔE3^ cells exhibited significantly higher sensitivity to CX5461 compared to the sensitivity exhibited by the WT cells (IC50: WT/WT 16.1 nmol/l, ΔE3/ΔE3 7.3 nmol/l) as expected (Fig. 6C). CX5461 is anticipated as a drug with selective lethality in BRCA1/2-deficient tumors, and phase I clinical trials are being conducted for patients with BRCA1/2-deficient tumors^38^. Therefore, we tested whether the HERC2 dysfunction further sensitized the cells with BRCA deficiency to CX5461. HeLa-shHERC2, HCT116-shHERC2, and wild-type and HERC2^ΔE3/ΔE3^ HCT116 cells were transfected with either control or BRCA1-specific siRNA. The effect of siRNA- and/or Dox-induced knockdown of BRCA1 and HERC2 was confirmed via immunoblotting (Fig. 6D,E). Cells were then exposed to CX5461, and clonogenic survival assays were performed. We observed that BRCA1 deficiency sensitized the cells to CX5461 in the HERC2 intact cells, which was in concordance with the findings of a previous report^38^ (Supplementary Figure S11). Interestingly, HERC2 dysfunction salvaged, instead of sensitized, the BRCA1-depleted cells to CX-5461 for all cell lines, thereby contradicting our expectations (Fig. 6F–H). Altogether, these results indicate that HERC2 function is critical for the sensitization of cells to CX5461.

## Discussion

Ribosome biogenesis, in particular rRNA transcription, has recently gained attention as a novel target for cancer treatment, represented by Pol I inhibitors CX-5461^37^, CX-3543^41^, and BMH-21^42,43^. BLM and WRN are the two major DNA helicases involved in the resolution of DNA secondary structures and play critical roles in the transcriptional activity of Pol I^12–16,24–26^. These helicases are particularly important for the maintenance of regions that are enriched with G4 motifs such as telomeres, promoters, and nucleolar rDNA^6–12^, where the requirement for the helicase-mediated DNA resolution is prominent. In this study, we report that HERC2 regulates the nucleolar localization of helicases and regulates the effect of CX-5461 on pre-rRNA transcription and cell viability.

HERC2 is a nuclear-cytoplasmic shuttle protein, found in the cytoplasm and the nucleus^44,45^. It co-localizes at foci with replication fork complex proteins in the nucleus^27,36^. Here, we demonstrated that in addition to the nuclear foci, HERC2 localized in the nucleolus with fibrillarin and Pol I subunit, RPA19, along with BLM and WRN. Numerous studies have indicated the crucial roles of BLM and WRN in Pol I activity and subsequent ribosome assembly. BLM interacts with Pol I and is required for efficient rRNA transcription possibly through its helicase activity, as BLM can unwind GC-rich rDNA-like substrates that form in the nucleolus and can normally inhibit progression of the Pol I complex^24^. WRN also acts as a component of the Pol I complex. Fibroblasts from Werner syndrome exhibited decreased levels of rRNA transcription that could be rescued by exogenous expression of the wild-type WRN, but the mutant WRN, which lacks an ability to localize in the nucleolus^26^, did not exhibit such a rescue mechanism. These observations indicate that BLM and WRN and their nucleolar localization are critical for rRNA transcription. Since HERC2 is required for the proper nucleolar localization of these helicases, it is reasonable to expect that HERC2 deficiency would affect the Pol I activity in rRNA transcription. However, HERC2 dysfunction on its own did not inhibit pre-rRNA transcription. The deficiency in BLM or WRN independently inhibits rRNA transcription^24–26^; however, the lack of these helicases in the presence of HERC2 deficiency exhibited differential effects as compared to that observed in simple helicase inhibition. However, HERC2 deficiency has some impact on the rRNA transcription. In support of this observation, both HERC2-depleted cells and cells lacking HERC2 E3 activity showed an increase in the suppressive effect of CX-5461 on pre-rRNA transcription, but not Pol II-mediated c-Myc transcription. HERC2 could be critical for the localization and subsequent G4 resolution activity of BLM and WRN to execute effective rRNA transcription that is essential under CX-5461-induced ribosome stress conditions.

The nucleolus plays a crucial role in stress response in addition to ribosome biogenesis. It functions by the sequestration and release of nucleolar proteins^32^. In response to DNA damage, WRN disseminates from the nucleolus towards the nucleoplasm to function at the site of DNA damage^46^. However, it is elusive whether BLM is sequestrated in the nucleolus and released after replication stress, as there have been conflicting studies that have either demonstrated release from the nucleolus^35^ or unaffected retention in the nucleolus^24^, post HU treatment. Our results were consistent with the findings of the former reports. One interpretation of the discrepancy is that the durations for the HU treatment were relatively longer in the experiments, resulting in unaffected BLM localization in the nucleolus, with 16 h, whereas we performed the analysis after 4 h of treatment. Time-course analyses showed that BLM was released from the nucleolus after 4 h and relocalized at stalled replication forks with RPA in approximately 10 to 16 h. In HERC2-deficient cells, however, the relocalization of BLM was abrogated which was accompanied by the lack of prerequisite nucleolar BLM. This is consistent with our previous hypothesis that HERC2 functions, in part, as a cellular stock for the stress-responsive proteins^27^. It is also possible that HERC2-dependent function of RNF8 and RNF168 may contribute to the localization of BLM to the stalled replication fork^30,31^, although they did not participate in the HERC2-dependent nucleolar localization of BLM and WRN.

Given the G4-enriched structure with the highest frequency of transcription that creates conflicts with replication, rDNA genes are vulnerable to DNA damage, and spontaneous alterations in the rDNA gene subsequently leads to cancer development^47^. On the other hand, such vulnerability is a candidate target for the treatment of cancer, where there is an increased demand for protein synthesis. Accordingly, there are numerous ongoing preclinical and clinical studies of Pol I inhibitors, especially CX-5461, for a variety of tumors including hematologic cancers^48–52^, small cell lung cancer^53^, prostate cancer^54^, ovarian cancer^55,56^, and BRCA1/2 deficient breast and ovarian cancer^38^. CX-5461 is a selective inhibitor of Pol I-mediated rDNA transcription. It functions by disrupting the association of Pol I transcription factor SL1 with the rDNA promotor ^37^. Additionally, CX-5461 binds and stabilizes the G4 structure in vitro and impedes the progression of DNA replication accompanied by G4 accumulation in vivo^38^. As HERC2 dysfunction sensitizes cells to other G4 stabilizers^27,29^, we tested whether this phenomenon was the same for CX-5461. However, it was only observed in HERC2^ΔE3/ΔE3^ cells, and not for cells with HERC2 knockdown, despite these cells possessing less ability for rRNA transcription and G4 resolution. The mechanism underlying this particular discrepancy remains unclear. This may be attributed to decreased efficiency of HERC2 knockdown as compared to knockout, efficacy of CX-5461 as a G4 stabilizer, or the type of G4 stabilized by CX-5461. Noteworthy, previous screening of CX-5461 response using a panel of DNA damage response mutant strains of *C. elegans* demonstrated that *him-6* and *wrn-1* (orthologues of *BLM* and *WRN*) genes were not responsible for CX-5461 sensitivity, whereas it identified other genes involved in replication-associated repair and resolution of G4 structures including *mus-81*, *rfs-1*, *polq-1*, *helq-1*, and *rtel-1*, as genes responsible for CX-5461 sensitivity^38^. In addition, recent studies indicated that CX-5461 mediates its cytotoxic effects through trapping of topoisomerase II^39,40^. The level of this activity could contribute the effects of CX-5461 different from other G4 stabilizers on the viability of HERC2-knockdown cells.

The G4-stabilizing activity of CX-5461 has recently gained increased attention since it demonstrates synthetic lethal effect with homologous recombination failure due to BRCA1/2 deficiency^38^, which is the case for other G4 stabilizers including pyridostatin^57,58^. An unbiased genome-wide study that identified genes that promote cell death when silenced by shRNA in the presence of G4-stabilizers validated *BRCA1/2* and *HERC2* as responsible genes^29^. Thus, we conducted clonogenic survival assays to ensure the synergetic or additive effect of double inhibition of BRCA1 and HERC2 on the sensitivity to CX-5461. However, confounding the prediction, inhibition of HERC2 or its E3 activity reversed, rather than enhanced, the increased cell death mediated by BRCA1 depletion and CX-5461. The mechanisms underlying this phenomenon remains unknown. However, since HERC2 is downregulated in numerous cancers^27^, these findings may be of clinical significance considering the beneficial effects of CX-5461 in cancer treatments.

## Materials and methods

### Cell lines and culture conditions

HeLa and HCT116 cells were obtained from ATCC with authentication and stored in liquid nitrogen or cultured according to the supplier’s instructions for less than 20 passages. HeLa and HCT116 cells, stably expressing HERC2-specific shRNA (5′-GAAGGTGGCTGTTCACTCA-3′ or #2: 5′-GGAAAGCACTGGATTCGTT-3′) in a Dox-inducible manner (HeLa-shHERC2 and HCT116-shHERC2), and HCT116 cells lacking the HERC2 catalytic ubiquitin-binding site (HCT116-HERC2^ΔE3/ΔE3^), as a result of CRISPR/Cas9-mediated insertion of the stop codon at E4758, were established as per previously described methods^27^. For the knockdown of HERC2 expression, HeLa-shHERC2 or HCT116-shHERC2 cells were treated with 1 µg/ml Dox for 48 h and then used for subsequent experiments. To induce replication stress, cells were incubated with 1 mM mM HU for 4 h or the indicated times to detect the disappearance of BLM from nucleoli or BLM foci formation, respectively. Chemical agents used were HU (Sigma-Aldrich) and CX-5461 (Selleck).

### Antibodies

Rabbit polyclonal antibody against the C-terminal region of HERC2, generated against recombinant HERC2 protein (residues 4389–4834), was established as per previously described methods^27^. The commercially available antibodies used in the study were: rabbit polyclonal antibodies against BLM (Bethyl Laboratories, A300-110A), WRN (Bethyl Laboratories, A300-239A), BRCA1 (Santa Cruz Biotechnology, C20), mouse monoclonal antibodies against HERC2 (epitope 1781–1974, BD Biosciences, 17), NPM1 (Zymed Laboratories Inc), FBL (Abcam, 38F3), RPA194 (Santa Cruz Biotechnology, C1), RPA2 (Calbiochem, RPA34-20 NA19L), and α-tubulin (Neomarkers, DM1A, MS-581-P).

### Immunoblotting

Total cell lysates were prepared using RIPA buffer (50 mM Tris–HCl [pH 7.5], 150 mM NaCl, 0.1% SDS, 0.5% sodium deoxycholate, 1% Triton X 100, 1 mM dithiothreitol, 1 mM NaVO3, 1 mM PMSF, 2 µg/ml aprotinin, 2 µg/ml leupeptin, 10 µg/ml trypsin inhibitor, and 150 µg/ml benzamidine). Immunoblotting was performed as per methods described previously^59^.

### siRNAs and transfections

siRNA oligonucleotides targeting BRCA1 (CUAGAAAUCUGUUGCUAUG, D-003461–08) and the non-targeting control siRNA (D-001210-05) were purchased from Dharmacon. RNA duplexes (final concentration, 10 nM) were transfected into the cells using Lipofectamine RNAiMAX (Invitrogen) and analyzed 48 h post transfection.

### Immunofluorescence microscopy

Indirect immunofluorescence labeling of cells and fluorescence detection were performed as per methods described previously^60^ with the following modifications. For detection of nucleolar proteins, proliferating cells were fixed with 3% formalin for 20 min and permeabilized with 0.2% Triton X-100 for 20 min. For detection of nuclear foci of BLM and RPA, proliferating cells were pre-extracted with a buffer containing 20 mM HEPES (pH 7.5), 20 mM NaCl, 5 mM MgCl2, and 0.5% IGEPAL (A-630; Sigma-Aldrich) supplemented with proteinase inhibitors for 20 min on ice, and fixed with 2% formalin prepared in PBS for 20 min at room temperature. For detection of HERC2 in combination with other proteins, cells were fixed and permeabilized with cold methanol and acetone, respectively, as per methods described previously^36^. Cells were then washed, blocked with 3% goat serum and 0.1% Triton X-100, and labeled with primary and fluorescent-labeled secondary antibodies. The slides were mounted with the ProLong Gold Antifade Mountant with DAPI (Invitrogen) and examined with a confocal laser-scanning microscope (LSM 510, Carl Zeiss, Germany). For the quantification of the population of cells positive for nucleolar staining, cells displaying at least one positive nucleolus were counted as positive. For the quantification of the population of cells positive for BLM nuclear foci, cells displaying more than ten BLM foci co-localizing with RPA2 were counted as positive. The specificity of antibodies against HERC2, BLM and WRN for immunostain was validated with siRNA-mediated depletion of target proteins.

### qRT-PCR

Cells were seeded in 6-well plates, and then incubated with 0.002 to 1.25 μmol/l of CX-5461 for 2 h the following day. HeLa-shHERC2 and HCT116-shHERC2 cells were untreated or treated with Dox for 48 h before plating, with continued addition of Dox until harvesting. Total RNA from each sample was isolated using the RNeasy kit (QIAGEN) according to the manufacturer's instructions. Relative levels of pre-rRNA, c-Myc mRNA, and β-actin mRNA were measured using One Step TB Green (TaKaRa) analyzed on the StepOnePlus Real-Time PCR System (Applied Biosystems) using the following primers. pre-rRNA: forward primer, 5′-CCGCGCTCTACCTTACCTACCT-3′; reverse primer, 5′-GCATGGCTTAATCTTTGAGACAAG-3′; c-Myc mRNA: forward primer, 5′-CCTGGTGCTCCATGAGGAGAC-3′; reverse primer, 5′-CAGACTCTGACCTTTTGCCAGG-3′; β-actin mRNA: forward primer, 5′-GACCTCTATGCCAACACAGT-3′; reverse primer, 5′-AGTACTTGCGCTCAGGAGGA-3′. Each reaction was performed in triplicate and data were analyzed according to the comparative Ct method and were normalized using β-actin expression in each sample.

### Clonogenic survival assay

Cells were seeded at a density of 500 cells/well in 6-well plates and indicated concentrations of CX-5461 were added after 6 h. For cells that were subjected to siRNA treatments, a density of 6 × 10^4^ cells/well was seeded. After 24 h of incubation with CX-5461, the cells were washed and further cultured in fresh medium without CX-5461 for 14 days. The cells were then fixed and stained using crystal violet. The colonies were scanned and counted using the ImageQuant LAS-4000 instrument (GE Healthcare). HeLa-shHERC2 and HCT116-shHERC2 cells were untreated or treated with Dox for 48 h before plating, with continued addition of Dox to the medium until the fixation procedure.

## Supplementary Information

Supplementary Figures.

## Data Availability

The datasets generated and/or analyzed during the current study are available from the corresponding author on reasonable request.
