# Supplementary material for: HERC2 inactivation abrogates nucleolar localization of RecQ helicases BLM and WRN
Source: Sci Rep. 2021 Jan 11;11:360. doi: 10.1038/s41598-020-79715-y (PMC7801386; doi:10.1038/s41598-020-79715-y)
Supplement: Supplementary file 1 — Supplementary Figures. [file 41598_2020_79715_MOESM1_ESM.pdf]

Supplementary figures

HERC2 inactivation abrogates nucleolar localization of RecQ helicases BLM and WRN

Mingzhang Zhu, Wenwen Wu, Yukiko Togashi, Weixin Liang, Yasuo Miyoshi and Tomohiko Ohta

Supplemental information includes 12 figures.

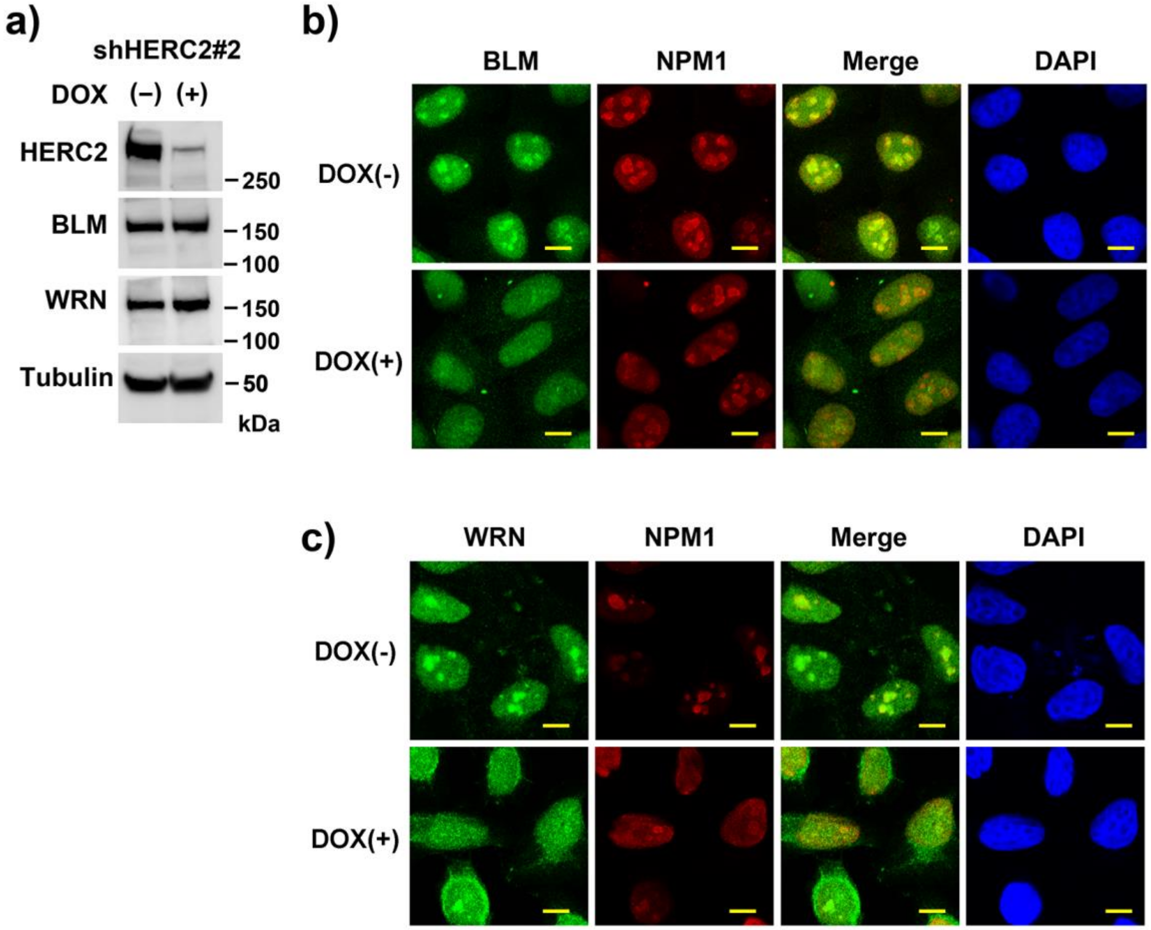

Supplementary Figure S1 (related to Figure 1 and 2)

Nucleolar localization of BLM and WRN in HeLa cells with a different shRNA against shHERC2. HeLa-shHERC2#2 cells with Dox-mediated induction or without induction and immunoblotting (a) or immunostaining with antibodies against BLM (b), WRN (c), and NPM1 (b and c) was performed. The nuclei were counter stained with DAPI. Scale bar, 10 μm.

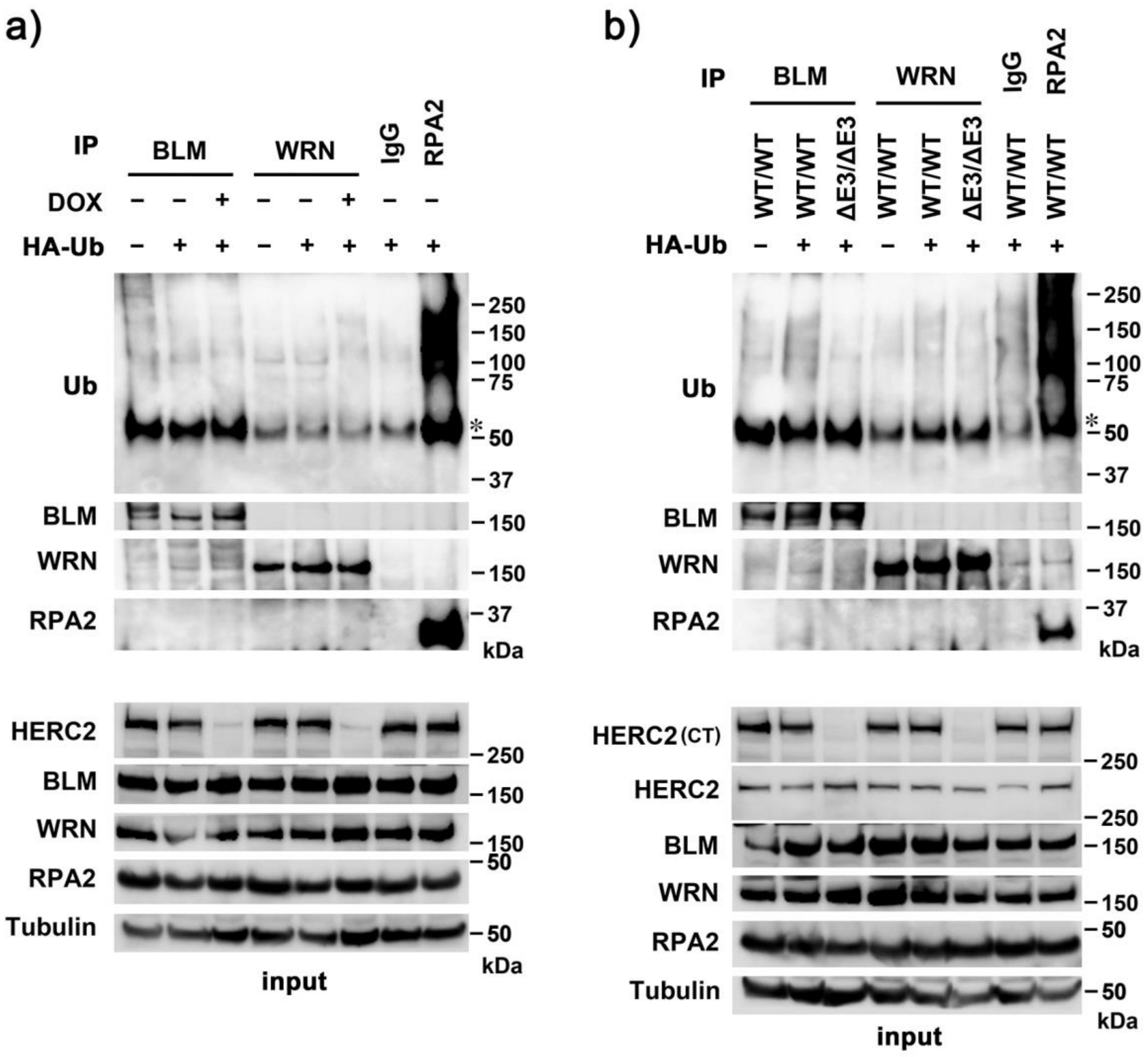

Supplementary Figure S2

Ubiquitination of endogenous BLM and WRN was not detected. (a) HeLa-shHERC2 cells were transfected with 6x tandem repeat HA-tagged ubiquitin (HA-Ub) as indicated, with Dox-mediated induction or without induction, treated with MG132, and subjected to immunoprecipitation in denatured condition with control IgG or indicated antibodies including anti-RPA2 antibody as a positive control, followed by immunoblotting with the indicated antibodies. Inputs were also loaded. (b) Wild type and HERC2$^{\Delta E3/\Delta E3}$ HCT116 cells were transfected with HA-Ub as indicated, treated with MG132, and subjected to immunoprecipitation and immunoblotting as shown in (a).

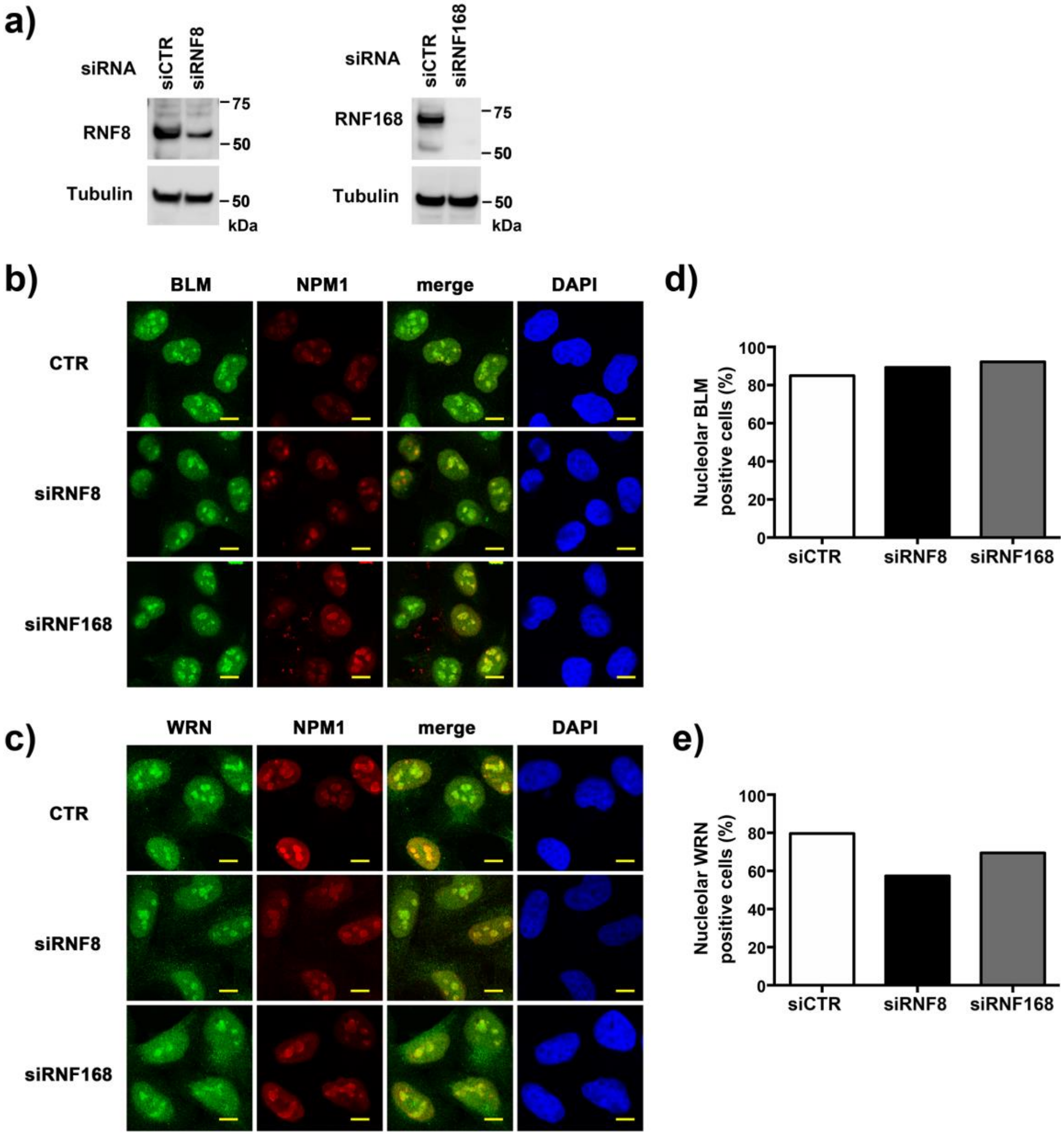

Supplementary Figure S3

Nucleolar localization of BLM and WRN is unaffected by depletion of RNF8 or RNF168. (a to c) HeLa cells were transfected with control (siCTR), RNF8-, or RNF168-specific siRNA and subjected to immunoblotting (a) or immunostaining with antibodies against BLM (b), WRN (c) and NPM1. The nuclei were counter stained with DAPI. Scale bar, 10 μm. (d and e) Quantifications of the nucleolar BLM-positive (d) or WRN-positive (e) cells, with each quantification based on more than 100 cells.

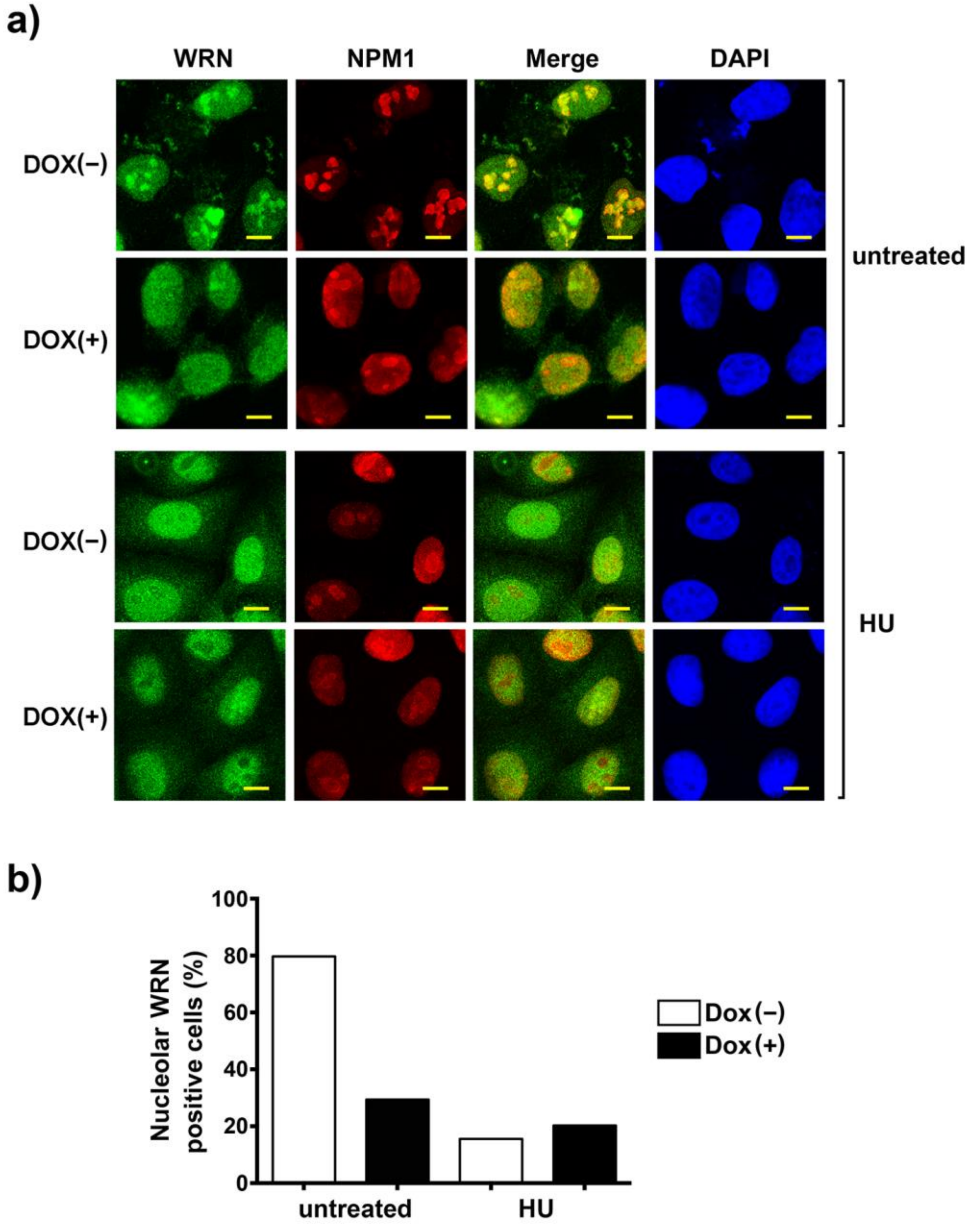

Supplementary Figure S4 (related to Figure 3)

WRN disappears from the nucleolus in response to replication stress. HeLa-shHERC2 cells with Dox-mediated induction or without induction, untreated or treated with 1mM HU for 4 hours, and immunostained with antibodies against WRN and NPM1. Scale bar, 10 μm. (b) Quantification of the nucleolar WRN-positive cells with or without HU treatment.

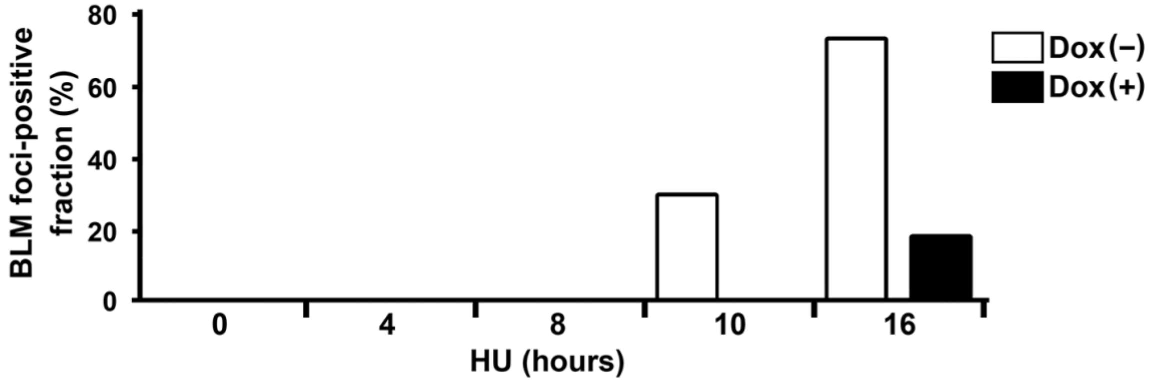

Supplementary Figure S5 (related to Figure 3)

Time-course experiments showing BLM nuclear foci formation post HU treatment. HeLa-shHERC2 cells with Dox-mediated induction or without induction, treated with 1mM HU for the indicated times, and immunostained with antibodies against BLM and RPA2. Quantification of the cells displaying more than ten BLM foci co-localizing with RPA2 at each time points is shown.

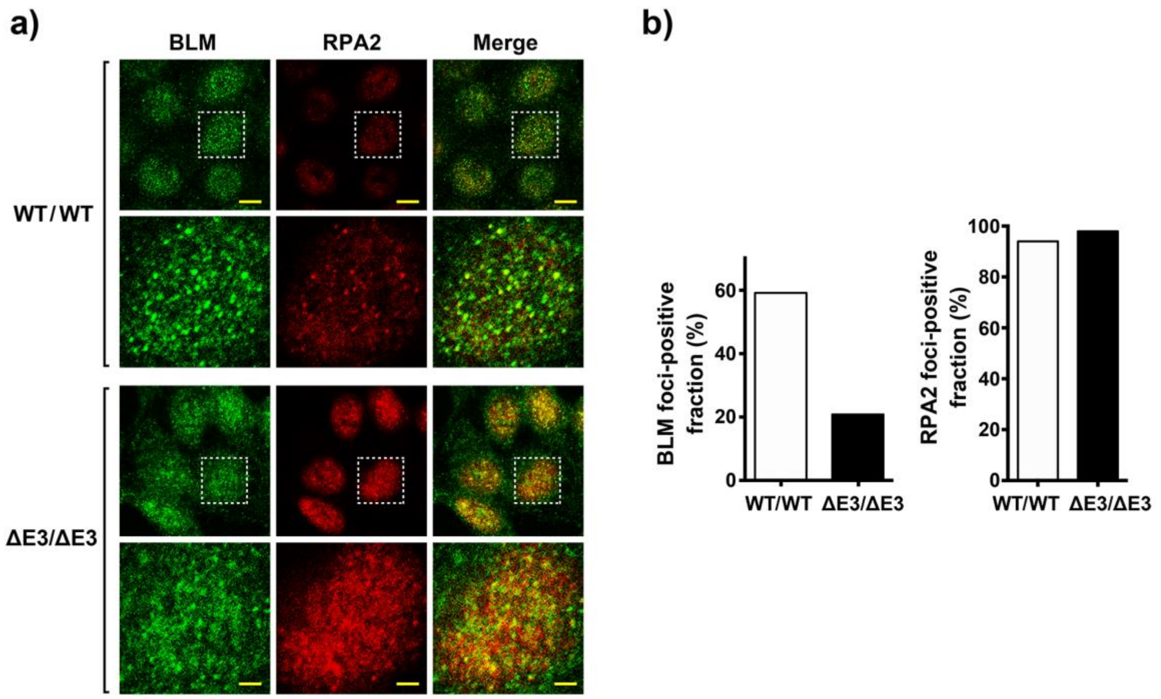

Supplementary Figure S6 (related to Figure 3)

Subnuclear localization of BLM and RPA2 in cells with HERC2 E3 domain deletion in response to replication stress. (a) WT or HERC2$^{\Delta E3/\Delta E3}$ cells were treated with 1 mM HU for 16 hours, and immunostained with the indicated antibodies. One representative nucleus (dashed line) in the upper panels has been magnified in the lower panels. Colocalization of BLM and RPA2 is shown in the merged figure. Scale bar, 10 μm. (b) Quantification of the cells displaying more than ten BLM foci co-localizing with discrete RPA2 foci (left panel) or discrete RPA2 foci (right panel) is shown.

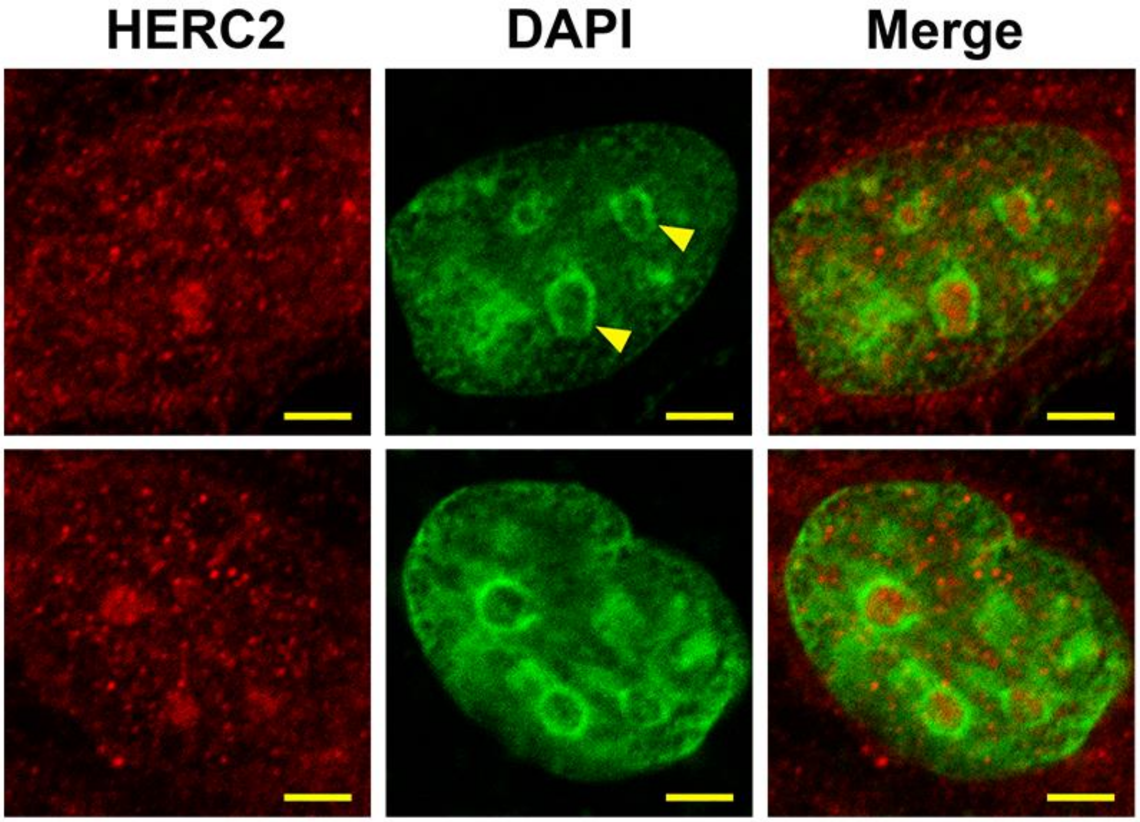

Supplementary Figure S7 (related to Figure 4)

Subnuclear localization of HERC2. Growing Hela cells were immunostained with HERC2 antibody. The nuclei were counter stained with DAPI. Two representative nucleoli were indicated by arrowheads. Scale bar, 4 μm.

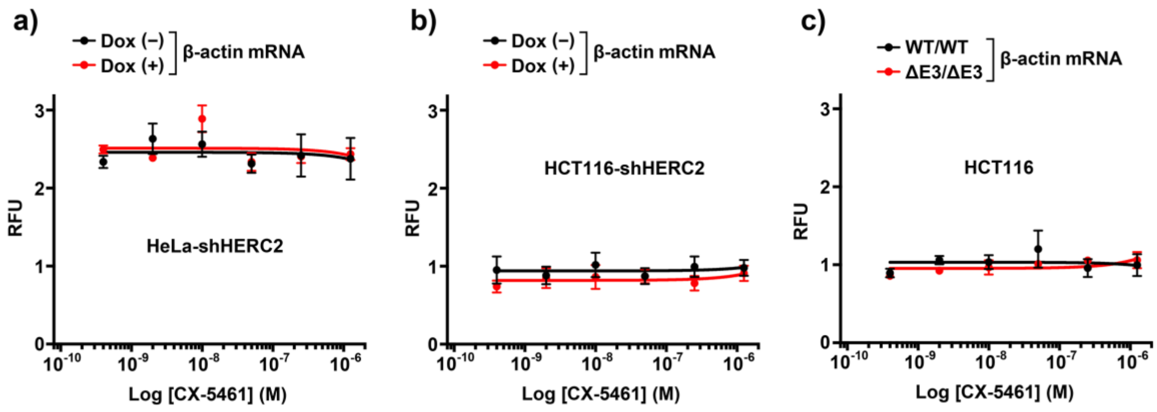

Supplementary Figure S8 (related to Figure 5)

β-actin mRNA expression of the cells treated with CX-5461. HeLa-shHERC2 (a) or HCT116-shHERC2 (b) cells untreated (black) or treated with Dox (red), and either wild-type (black) or HERC2$^{\Delta E3/\Delta E3}$ (red) HCT116 cells (c) were incubated with the indicated dose of CX5461. The level of β-actin mRNA expression was analyzed via qRT-PCR. Data are shown as the means ± S.D. of three independent experiments. RFU: relative fluorescence units.

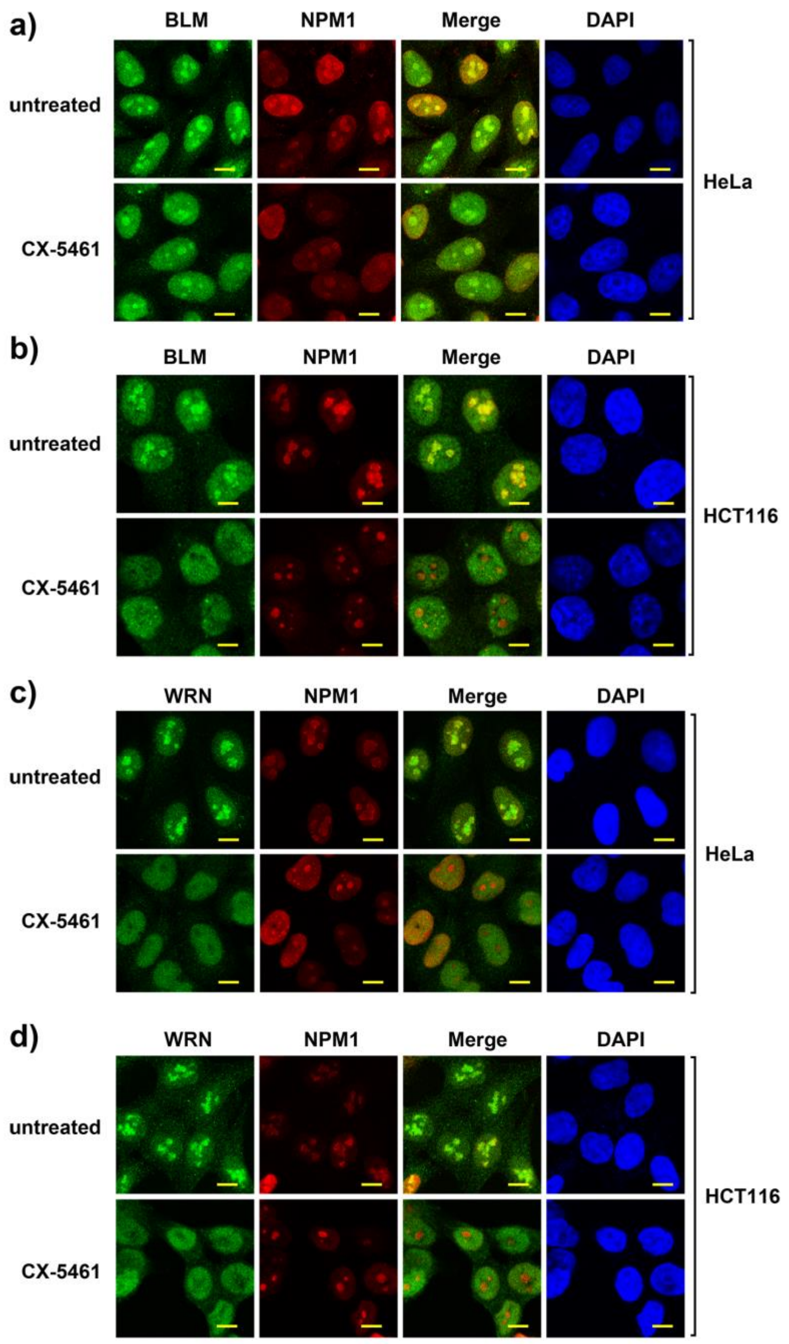

Supplementary Figure S9 (related to Figure 5)

BLM in HCT116 cells, and WRN in HeLa and HCT116 cells disappeared from the nucleolus post treatment with CX-5461. HeLa (a and c) or HCT116 (b and d) cells were untreated or treated with 1.25 μM CX-5461 for 4 hours, and immunostained with antibodies against BLM (a and b), WRN (c and d), and NPM1. Scale bar, 10 μm.

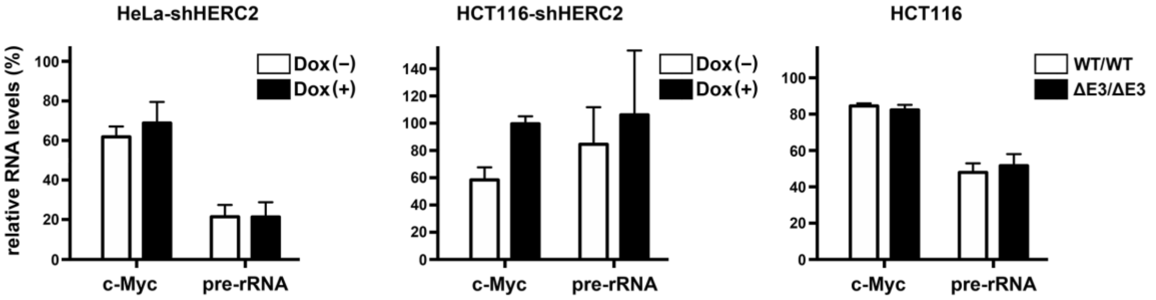

Supplementary Figure S10 (related to Figure 5)

HERC2 dysfunction alone does not inhibit pre-rRNA transcription. HeLa-shHERC2 or HCT116-shHERC2 cells untreated or treated with Dox, and wild-type or HERC2$^{\Delta E3/\Delta E3}$ HCT116 cells were analyzed for the level of pre-rRNA and c-Myc mRNA transcription via qRT-PCR, and normalized with β-actin mRNA expression. Data are represented as the mean ± S.D for three independent experiments.

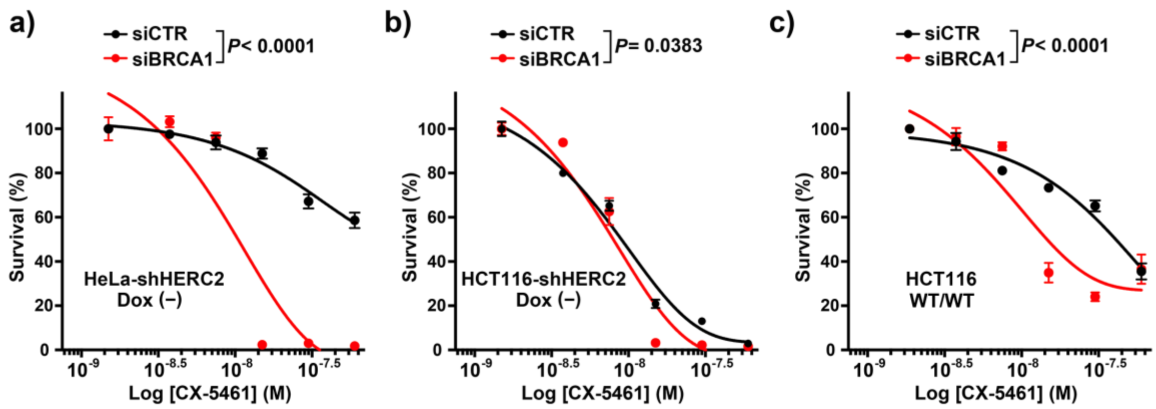

Supplementary Figure S11 (related to Figure 6)

BRCA1 deficiency sensitizes cells to CX-5461. HeLa-shHERC2 (a), HCT116-shHERC2 (b), and wild-type HCT116 cells (c) were transfected with control or BRCA1-specific siRNA without Dox induction, and exposed to indicated doses of the CX5461 for 24 hours and analyzed for clonogenic survival after two weeks. The data are shown with the nonlinear regression fit curves of one phase decay (GraphPad Prism). Average ± S.E.M., normalized to cellular expression in the absence of these agents were derived from experiments performed in triplicate. *P*-values of interactions were calculated using two-way ANOVA. The concentration that inhibited 50% of the colonies (IC50 value) was as follows: (a) siCTR: 74.2 nmol/L, siBRCA1: 10.3 nmol/L, (b) siCTR: 9.1 nmol/L, siBRCA1: 8.3 nmol/L, (c) siCTR: 40.7 nmol/L, siBRCA1: 16.9 nmol/L.

Fig. 1a

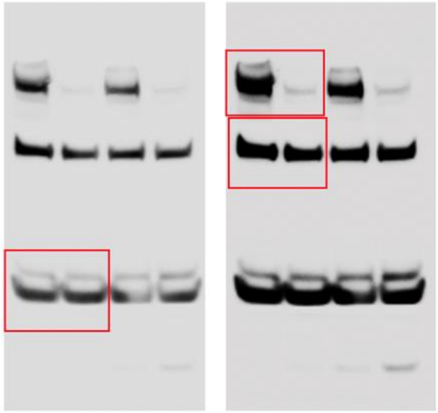

Fig. 1d

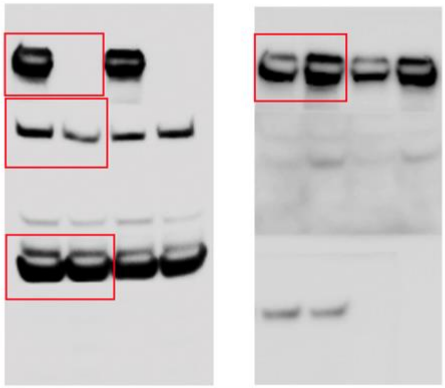

Fig. 2a & d

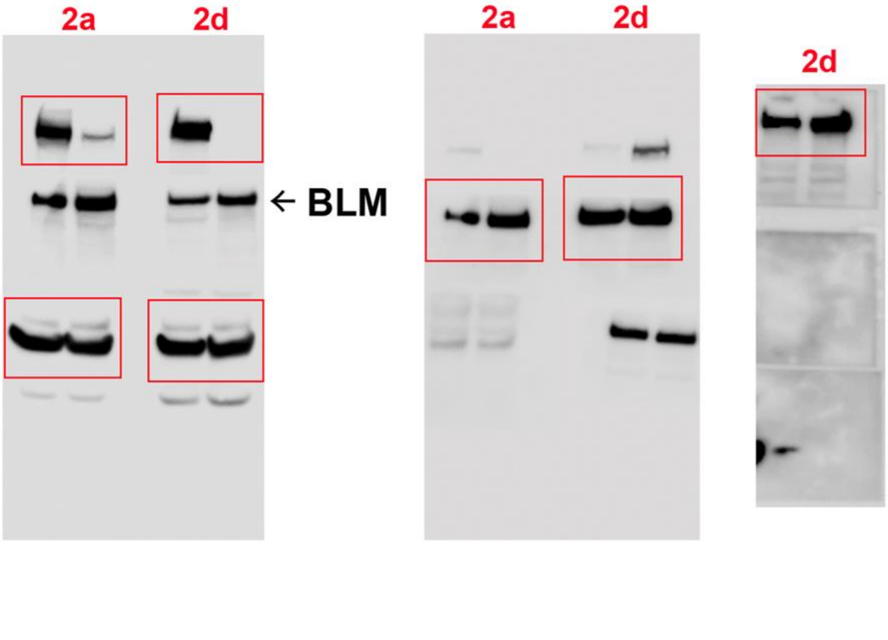

Fig. 6d

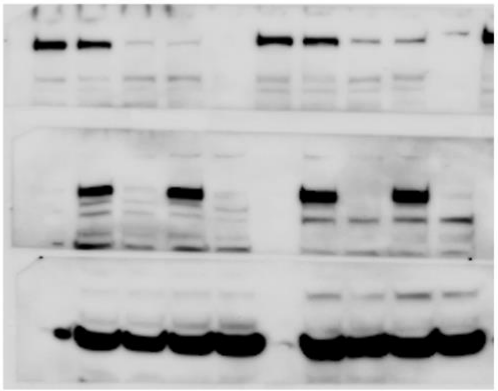

Fig. 6e

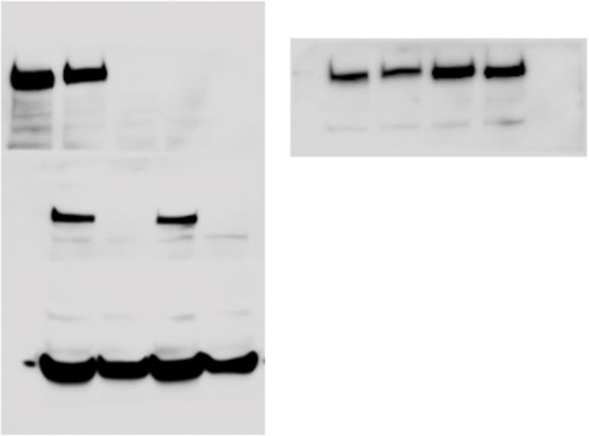

Fig. S1a

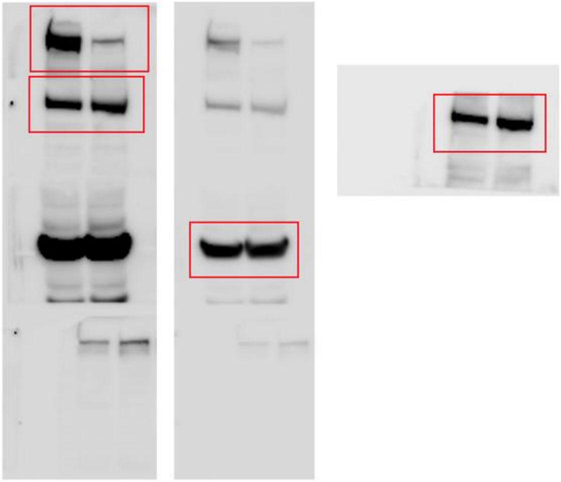

Fig. S2a & b (IP)

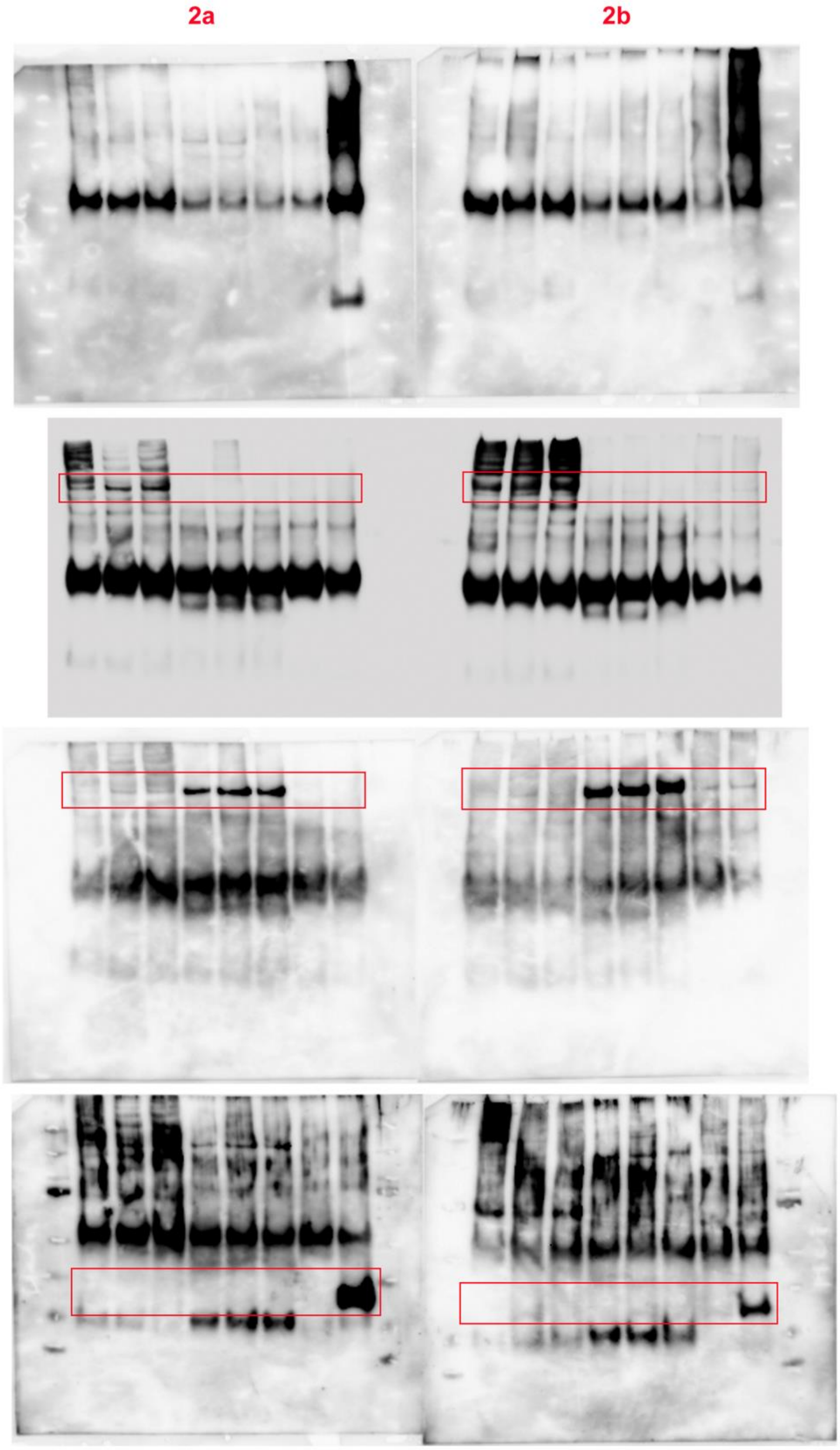

Fig. S2a & b (input)

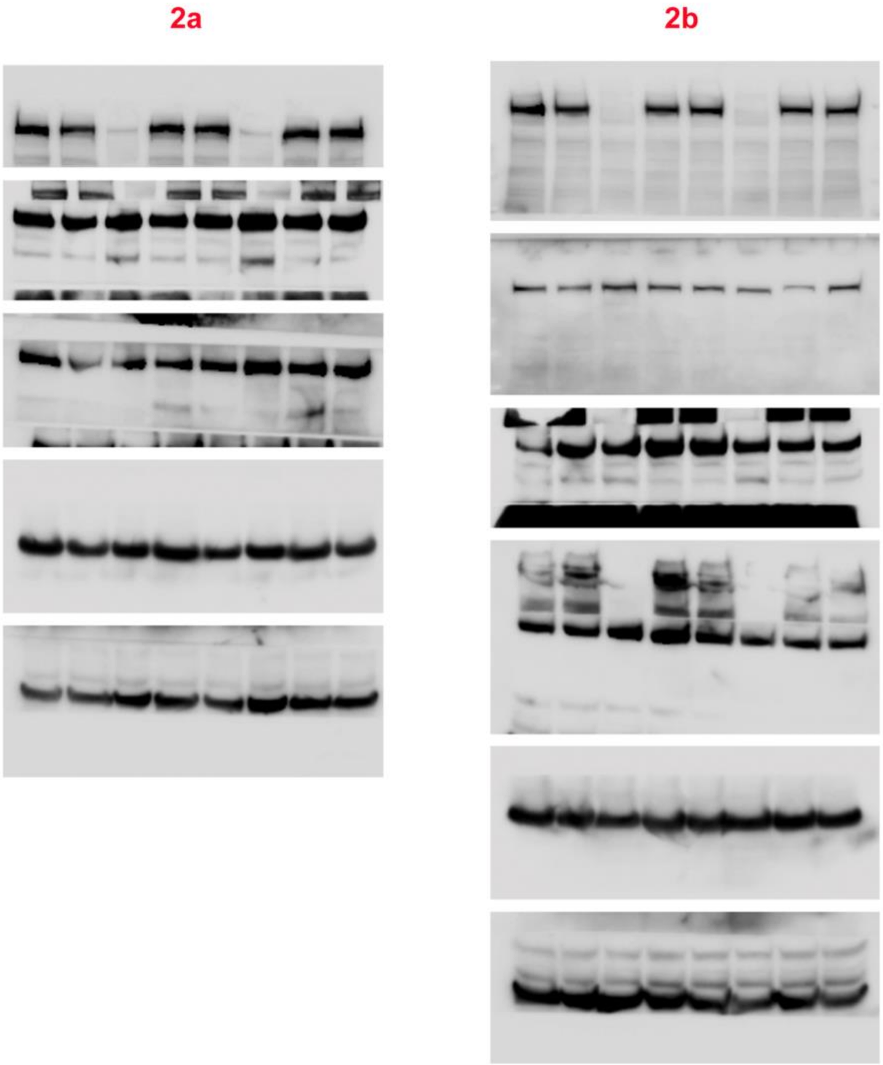

Fig. S3a

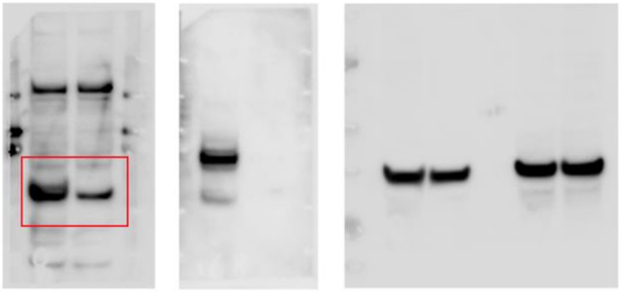

Supplementary Figure S12
Uncropped images of gels used in this study.
